# Is JmjC Oxygenase Catalysis Limited to Demethylation?**

**DOI:** 10.1002/anie.201303282

**Published:** 2013-06-20

**Authors:** Richard J Hopkinson, Louise J Walport, Martin Münzel, Nathan R Rose, Tristan J Smart, Akane Kawamura, Timothy D W Claridge, Christopher J Schofield

**Affiliations:** Chemistry Research Laboratory, University of Oxford12 Mansfield Road, Oxford, OX1 3TA (UK)

**Keywords:** demethylation, epigenetics, histone, hydroxylation, methyllysine

Histone modifications are of central importance in the regulation of transcription.^1^,^2^ Whilst histone acetylation is in general transcriptionally activating, histone lysine methylation can be activating or inhibitory depending on factors including the site and type of modification. Therefore, modulation of histone methylation is being pursued for the therapeutic regulation of gene expression.^3^–^6^

The JmjC family of *N*^*ε*^-methyllysine histone demethylases are ferrous iron and 2-oxoglutarate (2OG) oxygenases that are likely present in all animals.^7^–^9^ Unlike the flavin-dependent lysine-specific demethylases, the larger JmjC demethylase family accepts all 3 *N*^*ε*^-lysine methylation states. JmjC catalysis is proposed to proceed by means of hydroxylation to give a hemiaminal intermediate, which fragments to give the demethylated product and formaldehyde (Figure 1); however, to date the hemiaminal has not been observed.^10^,^11^ Although there have been studies on the sequence and methylation-state selectivities of JmjC enzymes,^12^–^15^ currently there are no reports on whether they can oxidize *N*-alkyl groups other than methyl or on their selectivity for the lysine side chain. Here we report substrate–selectivity studies with representative human JmjC demethylases, which reveal their potential to act on *N*-alkyl groups other than methyl, and to catalyze hydroxylation of groups other than *N*-methyl.

**Figure 1:**
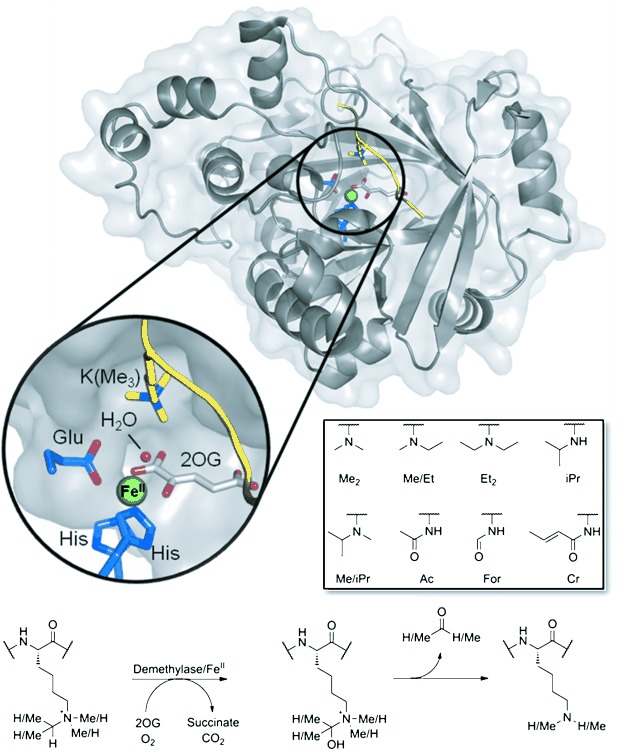
Top: View from a crystal structure of JMJD2A (KDM4A) (PDB ID: 2OQ6, with Ni substituted for Fe).^11^ Bottom: Mechanism of *N*^*ε*^-dealkylation catalyzed by JmjC demethylases. During demethylation, the hemiaminal intermediate is proposed to spontaneously fragment to give the demethylated lysine and HCHO. The structures of lysine analogues used in this work are boxed.

To investigate the selectivity of JmjC demethylases with respect to *N*-alkyl groups other than methyl, a set of histone H3 fragment peptides incorporating *N*^*ε*^-alkyl lysines at histone H3 lysine-9 and lysine-36 were synthesized and tested for reaction with representative JmjC demethylases JMJD2E (KDM4E), PHF8, and FBXL11 (KDM2A). These enzymes were chosen because of their different sequence and methylation-state selectivities: JMJD2E acts on H3 Lysine-9(Me_3_/Me_2_/Me_1_),^11^ PHF8 acts on H3 Lysine-9(Me_2_/Me_1_),^16^ and FBXL11 acts on H3 Lysine-36(Me_2_/Me_1_).^17^ We began by synthesizing *N*^*ε*^-diethyllysine (Lys(Et_2_)) analogues of the *N*^*ε*^-dimethyllysine substrates for the JmjC demethylases. However, in no case did we observe reaction under standard conditions when using MS-based assays. We then prepared the corresponding dialkylated *N*^*ε*^-methyl-*N*^*ε*^-ethyllysine (Lys(Me/Et)) analogues. In contrast to the Lys(Et_2_) analogues, clear mass shifts were observed when they were incubated with each of the enzymes JMJD2E, PHF8, and FBXL11 under appropriate conditions.

With JMJD2E, product peaks were observed with masses 28 Da and 42 Da lower than the Lys(Me/Et) substrate peptide, implying that both demethylation and de-ethylation occur to form the monomethylated and unalkylated lysines (Figure 2a). The assignments were supported by time course studies using MS and NMR analysis (Figure 2j and Figure S9); ^1^H NMR analyses indicate the formation of formaldehyde (Figure S10) and acetaldehyde (Figure 3a). The latter observation implies that de-ethylation occurs through hydroxylation adjacent to the *N*^*ε*^-ethyl lysyl amine, in an analogous manner to demethylation (Scheme 1).

**Figure 2:**
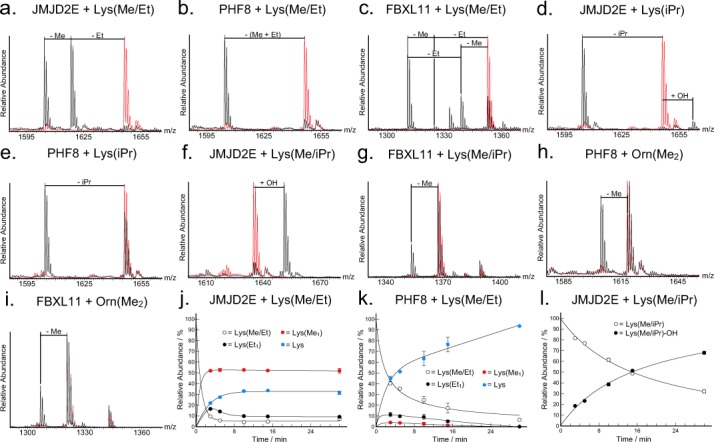
JmjC demethylases catalyze dealkylation and hydroxylation reactions. Mass spectra of incubations of a) ART-Lys(Me_3_)-QTAR-Lys(Me/Et)-STGGKA + JMJD2E, b) ART-Lys(Me_3_)-QTAR-Lys(Me/Et)-STGGKA + PHF8, c) PATGGV-Lys(Me/Et)-KPHRY + FBXL11, d) ART-Lys(Me_3_)-QTAR-Lys(*i*Pr)-STGGKA + JMJD2E, e) ART-Lys(Me_3_)-QTAR-Lys(*i*Pr)-STGGKA + PHF8, f) ART-Lys(Me_3_)-QTAR-Lys(Me/*i*Pr)-STGGKA + JMJD2E, g) PATGGV-Lys(Me/*i*Pr)-KPHRY + FBXL11, h) ART-Lys(Me_3_)-QTAR-Orn(Me_2_)-STGGKA + PHF8, and i) PATGGV-Orn(Me_2_)-KPHRY + FBXL11. Spectra of the corresponding substrate peptides are shown in red. MS timecourses for selected reactions: j) ART-Lys(Me_3_)-QTAR-Lys(Me/Et)-STGGKA + JMJD2E, k) ART-Lys(Me_3_)-QTAR-Lys(Me/Et)-STGGKA + PHF8, l) ART-Lys(Me_3_)-QTAR-Lys(Me/*i*Pr)-STGGKA + JMJD2E.

**Figure 3:**
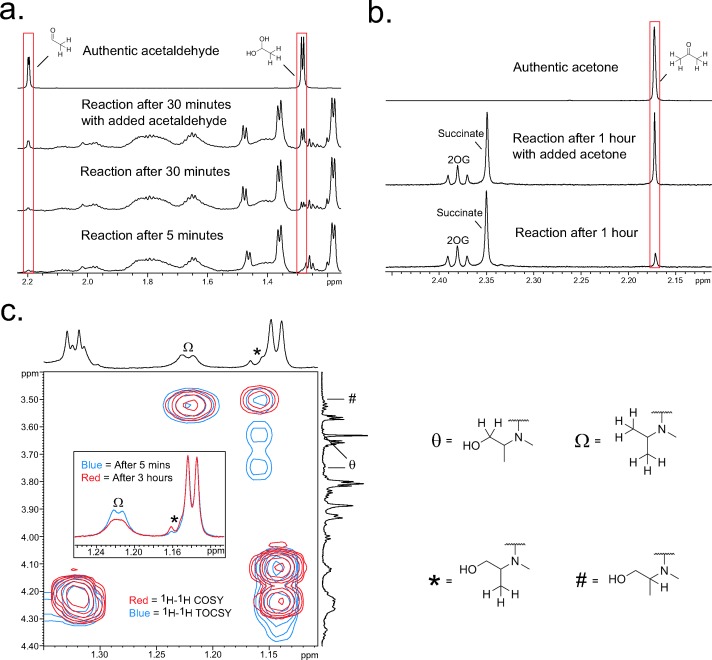
NMR analyses of reactions catalysed by JmjC demethylase. a) ^1^H NMR spectra of JMJD2E-catalyzed formation of acetaldehyde by reaction with ART-Lys(Me_3_)-QTAR-Lys(Me/Et)-STGGKA. b) ^1^H NMR spectra of JMJD2E-catalyzed formation of acetone by reaction with ART-Lys(Me_3_)-QTAR-Lys(*i*Pr)-STGGKA. c) ^1^H-^1^H COSY and TOCSY spectra of a sample containing ART-Lys(Me_3_)-QTAR-Lys(Me/*i*Pr)-STGGKA and JMJD2E after 1 hour of reaction. Correlations corresponding to the hydroxylated lysine fragment are highlighted.

**Scheme 1:**
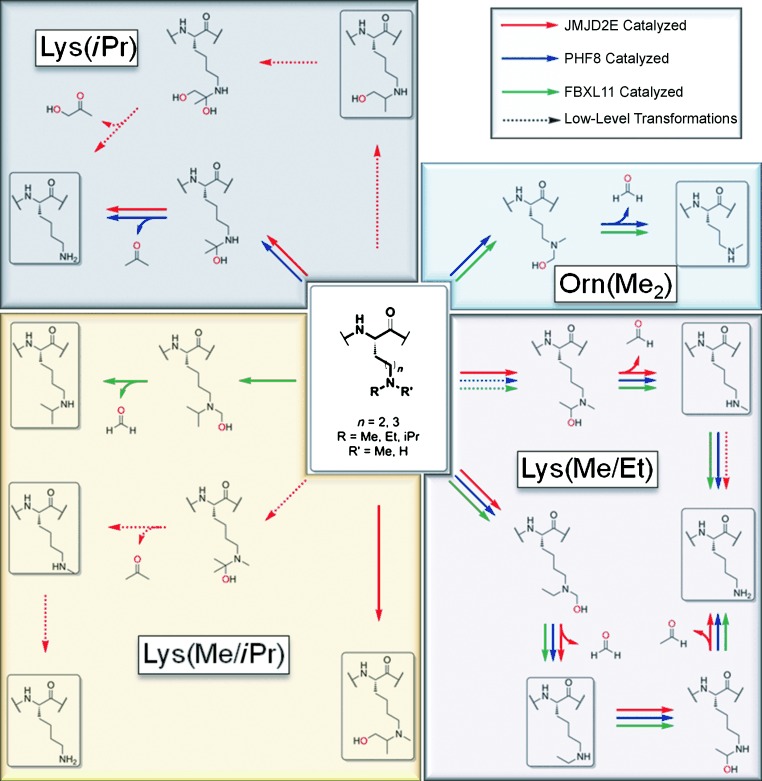
Summary of observed dealkylation/hydroxylation reactions catalyzed by JmjC demethylase.

The major product of the reaction of H3 Lysine-9(Me/Et) peptide with PHF8 was assigned as the unalkylated lysine peptide, indicating that both demethylation and de-ethylation occur under standard conditions (Figure 2b). However, no evidence for significant formation of *N*^*ε*^-monomethyllysine peptide (Lys(Me_1_)) was accrued, although the *N*^*ε*^-monoethyllysine peptide (Lys(Et_1_)) was detected in time course experiments (Figure 2k). Competition experiments with JMJD2E between the H3 Lysine-9(Me/Et) peptide (15-mer, ART-Lys(Me_3_)-QTAR-**Lys(Me/Et)**-STGGKA) and a closely analogous dimethyllysine peptide (14-mer, ART-Lys(Me_3_)-QTAR-**Lys(Me_2_)**-STGGK) provided evidence for the formation of the monomethyllysine peptide derived from the dimethyllysine peptide (14-mer), as well as for the monoethyllysine peptide and both 14-mer and 15-mer unalkylated peptides (from the Lys(Me_2_) and Lys(Me/Et) peptides, respectively; Figure S12). Therefore, it appears likely that de-ethylation of Lys(Me/Et) by PHF8 is disfavored relative to demethylation.

The results with PHF8 were largely mirrored in experiments with the H3 Lysine-36(Me/Et) peptide and FBXL11 (Figure 2c, and Figures S5 and S13), indicating similar reactivities for these two enzymes.

Overall, the results show that, when incorporated into appropriate peptide sequences, Lys(Me/Et) is a substrate for JMJD2E, PHF8, and FBXL11, producing monomethylated/monoethylated and unalkylated products. The tri-(JMJD2E) and di-(PHF8 and FBXL11) *N*^*ε*^-methyl demethylases display different preferences for the ethyl and methyl groups. We then investigated reactions of the H3 sequences containing *N*^*ε*^-isopropyllysine at Lysine-9 and Lysine-36 (Lys(*i*Pr)). With JMJD2E, the H3 Lysine-9(*i*Pr) peptide was observed to undergo loss of the isopropyl group to form the unalkylated lysine species (Figure 2d), as supported by ^1^H NMR analyses (Figure S14). MS experiments also revealed the formation of a low-level species with a mass 16 Da greater than that of the substrate peptide. Although its relatively low concentration precluded detailed characterization, it is likely that this product results from hydroxylation on an isopropyl CH_3_ group. The ^1^H NMR spectrum of the reaction mixture after 60 min at 25°C displayed a singlet resonance at *δ*_H_=2.17 ppm, which was assigned to the methyl protons of acetone by both ^1^H and ^13^C chemical shift analysis and by addition of a standard (Figure 3b and Figure S16). A small singlet resonance was also observed at *δ*_H_=2.09 ppm, which was tentatively assigned to the methyl protons of α-hydroxyacetone by ^1^H chemical shift analysis and by the addition of a standard (Figure S15). Therefore, it is probable that hydroxylation (and subsequent de-isoproylation) at the isopropyl CH can occur after hydroxylation on the isopropyl CH_3_. Notably, the Lys(*i*Pr) substrate reacted more efficiently than the analogous Lys(Me_1_)-containing peptide (14-mer, ART-Lys(Me_3_)-QTAR-**Lys(Me_1_)**-STGGK) during competition experiments with JMJD2E (Figure S18a), implying that both active-site fit and/or bond strength may be factors in determining the relative efficiency of dealkylation. With PHF8, de-isopropylation of the H3 Lysine-9(*i*Pr) peptide was also observed (Figure 2e); however, no evidence for the formation of a hydroxylated product was obtained. Interestingly, in competition experiments between the Lys(*i*Pr) peptide and the 14-mer Lys(Me_1_) peptide (see above) with PHF8 the monomethyllysine substrate is preferred (Figure S18b), which contrasts with the analogous experiment with JMJD2E. No reaction was observed between the H3 Lysine-36(*i*Pr) containing peptide and FBXL11, revealing different selectivities across the tested demethylases with respect to the Lys(*i*Pr) residue. We then analysed the reactions of *N*^*ε*^-methyl-*N*^*ε*^-isopropyllysine-containing peptides (Lys(Me/*i*Pr)) with the demethylases. Significantly, the predominant product in samples of the H3 Lysine-9(Me/*i*Pr) peptide with JMJD2E possessed a mass 16 Da higher than the starting peptide, implying hydroxylation (Figure 2f). This assignment was supported by ^1^H NMR analyses (Figure 3c and Figure S19). Upon incubation under ^18^O_2_, MALDI-TOF analyses revealed formation of a species with a +18 Da mass shift (Figure S20), further supporting oxygenase-catalyzed hydroxylation. ^1^H-^1^H COSY and TOCSY NMR analyses imply that hydroxylation occurs on an isopropyl CH_3_ group (Figure 3c), that is, two carbon atoms from the ε-amine. However, evidence for loss of the isopropyl group (and subsequent loss of the methyl group) was apparent in the MALDI-TOF spectra at low levels, indicating that oxidation of the isopropyl group adjacent to the ε-amine also occurs (trace levels of acetone were also detected in the NMR experiments, Figure S19). No reaction was observed in samples containing the H3 Lysine-9(Me/*i*Pr) peptide and PHF8. However, the samples with FBXL11 showed apparent demethylation of the *N*^*ε*^-methyl group in MS and NMR analyses (Figure 2g and Figures S7 and S21). This observation suggests that the more bulky isopropyl group is preferentially orientated away from the catalytic iron in the FBXL11 active site; that is, the selectivity differs from that of JMJD2E, where hydroxylation is observed.

Reactions with alkylated ornithine derivatives were then investigated. The *N*^*δ*^-dimethylornithine peptide (H3 Ornithine-9(Me_2_)) did not react with JMJD2E; however, MS activity assays with this peptide and PHF8 showed apparent demethylation to form the corresponding *N*^*δ*^-monomethylornithine peptide (H3 Ornithine-9(Me_1_), Figure 2h and Figures S8a and S22). These findings demonstrate that PHF8 is able to accept shortened lysine analogues, whereas JMJD2E does not; this observation correlates with X-ray analyses of JMJD2A (a close homologue of JMJD2E) and PHF8 with their respective H3K9 substrates (PDB codes 2OQ6 and 3KV4 respectively, Figure S25).^15^,^16^ In these structures, the methylated amine side chain at H3K9 apparently penetrates deeper towards the catalytic iron in PHF8 than in JMJD2A, implying that the shorter ornithine substrate may still bind sufficiently near the iron for oxidation to occur. FBXL11 also catalyzes demethylation of *N*^*δ*^-dimethylornithine when incorporated in an H3 Lysine-36 peptide (Figure 2i); this observation is consistent with the closer structural similarity of FBXL11 to PHF8 than to JMJD2E. Finally, no reactions, at least to give sufficient amounts of material for characterization, were observed for *N*^*δ*^-diethylornithine peptides (Orn(Et_2_)) with the three demethylases. This observation suggests that the diethylamine moiety is too bulky to fit productively into the active sites of the three enzymes; there was some MS-based evidence that Orn(Et_2_) peptide is hydroxylated by JMJD2E, although the low level of conversion precluded characterization. The demethylases were also tested with three *N*-acylated lysines; *N*^*ε*^-formyllysine (Lys(For)), *N*^*ε*^-acetyllysine (Lys(Ac)), and *N*^*ε*^-crotonyllysine (Lys(Cr)), respectively. All of these modifying groups have been identified on histones in cells.^18^–^20^ No reactions were observed for any of the peptides with the demethylases, suggesting that the tested enzymes do not modify such species, at least not with the sequences/conditions tested.

The relative reaction proficiencies of the analogues found to be substrates were determined and compared (Table S1). For all three enzymes, Lys(Me/Et) was the preferred substrate analogue; however, all analogues were weaker substrates than the analogous Lys(Me_2_) peptides. Kinetic parameters for the Lys(Me/Et), Lys(*i*Pr), and Lys(Me/*i*Pr) peptides with JMJD2E were also determined. *K*_M_, *V*_max_, and *k*_cat_ values for the Lys(Me/Et) peptide were similar to those attained for the corresponding Lys(Me_2_) peptide (Table S2). The Lys(*i*Pr) and Lys(Me/*i*Pr) peptides showed higher *K*_M_ values (284.2 μm and 164.1 μm, respectively, relative to 29.6 μm and 42.7 μm for the Lys(Me_2_) and Lys(Me/Et) peptides, respectively; Table S2). Interestingly, the *V*_max_ and *k*_cat_ values for the Lys(*i*Pr) peptide (*V*_max_=0.152 μms^−1^, *k*_cat_=0.303 s^−1^) were higher than values for the other three peptides, although the Lys(*i*Pr) peptide is a weaker substrate than the Lys(Me/Et) and Lys(Me_2_) peptides in competition experiments. This could reflect faster product release for the reaction with the Lys(*i*Pr) peptide, or that oxidation of the weaker tertiary isopropyl C–H bond is faster than oxidation of the primary and secondary C–H bonds in the other substrates.

Overall, our results clearly indicate the potential of JmjC enzymes to act on *N*-alkyl groups other than methyl. They also imply substrate selectivity may be determined both by active-site fit and C–H bond strengths. The observation that in addition to catalyzing *N*-dealkylation, JMJD2E can catalyze formation of a stable alcohol strongly supports the proposal of a hemiaminal intermediate during demethylation, and a close evolutionary relationship between the JmjC demethylases and related hydroxylases. Factor inhibiting hypoxia inducible factor (FIH), a transcription factor hydroxylase closely related to the JmjC demethylases, has been found to catalyze hydroxylation not only of asparagine residues (as in its action on hypoxia inducible factor), but also on aspartate and histidine residues present in ankyrin repeat proteins.^21^,^22^ Thus, it is possible that the selectivity of the JmjC enzymes (including those with as yet unassigned functions) is even wider than suggested by our current work. Although the physiological significance of our results is unclear, it is known that high concentrations of the ethanol metabolite acetaldehyde can lead to nucleic acid alkylation,^23^,^24^ which can be potentially removed by 2OG-dependent nucleic acid demethylases.^25^ It is thus a possibility that protein *N*-ethylation, if it occurs, is removed by 2OG oxygenase catalysis.

The results also highlight variations in substrate specificities between JmjC demethylases. For example, hydroxylation of Lys(Me/*i*Pr) was only observed with JMJD2E, suggesting that of the tested enzymes, only JMJD2E is able to bind the substrate in an orientation amenable to oxidation on the isopropyl CH_3_. Indeed, JMJD2E is able to catalyze hydroxylation of methyl, ethyl, and isopropyl groups, and in the latter case, at both primary and tertiary C–H bonds. In contrast, PHF8 was only observed to perform de-ethylation and de-isopropylation reactions when the lysine substrate was monoalkylated, whereas FBXL11 appears to preferentially orientate bulkier substituents away from the catalytic iron. These differences in binding preferences may be helpful in the design of selective inhibitors.

## Notes

As a service to our authors and readers, this journal provides supporting information supplied by the authors. Such materials are peer reviewed and may be re-organized for online delivery, but are not copy-edited or typeset. Technical support issues arising from supporting information (other than missing files) should be addressed to the authors.
