# Supplementary material for: Is JmjC Oxygenase Catalysis Limited to Demethylation?
Source: Angew Chem Int Ed Engl. 2013 Jun 20;52(30):7709–13. doi: 10.1002/anie.201303282 (PMC3798130; doi:10.1002/anie.201303282)
Supplement: Supplementary file 1 [file anie0052-7709-sd1.pdf]

Angewandte Chemie
Eine Zeitschrift der Gesellschaft Deutscher Chemiker

Supporting Information
© Wiley-VCH 2013
69451 Weinheim, Germany

Is JmjC Oxygenase Catalysis Limited to Demethylation?**

*Richard J. Hopkinson, Louise J. Walport, Martin Münzel, Nathan R. Rose, Tristan J. Smart, Akane Kawamura, Timothy D. W. Claridge, and Christopher J. Schofield**

anie_201303282_sm_miscellaneous_information.pdf

Supporting Information

Synthesis of Lysine Analogues

General Procedures

Reactions were carried out in dried round-bottomed flasks with magnetic stirring in a fume-hood. Thin layer chromatography (TLC) was performed using Merck aluminium foil backed plates precoated with Kieselgel 60 F254. Visualisation of species on the plates was achieved using UV fluorescence (254 nm) or by staining with potassium permanganate. NMR experiments were recorded using either Bruker AVANCE AV400 or Bruker AVII500 spectrometers. NMR spectra were referenced to the solvent peak using the Bruker internal referencing procedure. Coupling constants (*J*) are reported to the nearest 0.5 Hz. The following abbreviations are used to describe multiplicities; s = singlet, d = doublet, t = triplet, m = multiplet, dd = double doublet, br s = broad singlet, app t = apparent triplet. High-resolution mass spectra were recorded using Micromass GCT spectrometers. Infrared spectra were recorded as solids using a Bruker Tensor 27 instrument. Absorptions are given in wavenumbers (cm^{-1}). Only peaks of interest are reported. Melting points were carried out using a Griffin apparatus. $[\alpha]_D$ values were collected using a Bellingham and Stanley ADP220 polarimeter. Characterisation of the protected lysine analogues is reported for samples at over 90 % purity, which were used directly for solid phase peptide synthesis. Numbering for the aromatic carbon atoms is shown below.

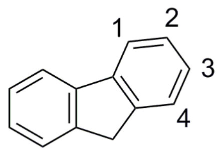

Numbering for the Fmoc carbons.

Fmoc-Lys(Boc)-OH, Fmoc-Lys(Ac)-OH and Fmoc-Lys(For)-OH were purchased from Bachem. Fmoc-Lys(Me,Boc)-OH was purchased from GM Biochem (Shanghai) Ltd. Other reagents were from Aldrich. Solvents were from either Aldrich or Rathburn Chemicals.

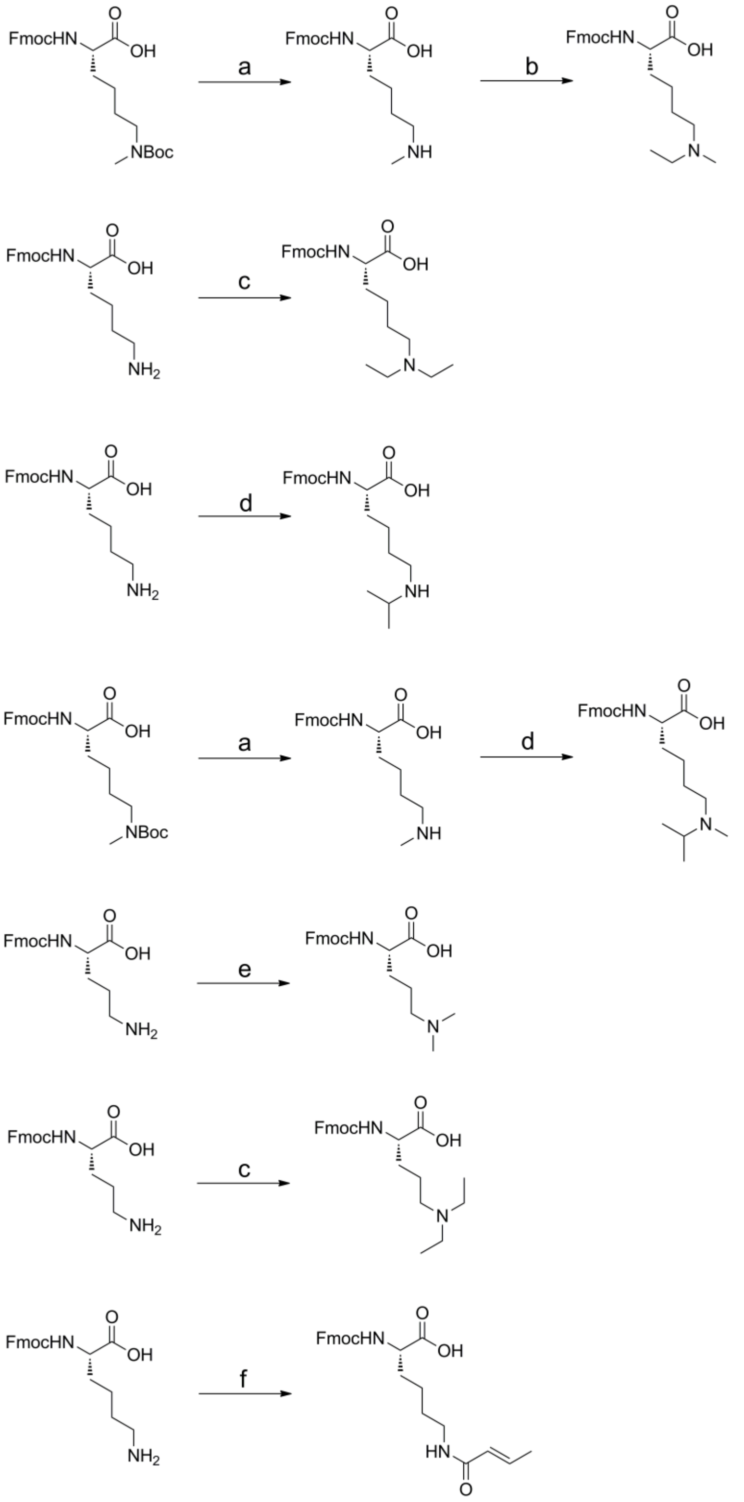

Scheme S1 Synthetic procedures for preparation of the N^{α}-Fmoc protected lysine analogues. a) CF_3COOH, triisopropylsilane; b) CH_3CHO (5 eq.), $NaBH_3CN$ (1.1 eq.), EtOH; c) CH_3CHO (5 eq.), $NaBH_3CN$ (2.1 eq.), EtOH; d) acetone (1.5 eq.), $NaBH_3CN$ (1.1 eq.), EtOH; e) HCHO (6 eq.), $NaBH_3CN$ (2.1 eq.), MeOH; f) crotonic anhydride (1.1 eq.), K_2CO_3 (2 eq.), 10:1 MeCN/DMF.

Characterisation of Lysine Analogues

a) Fmoc-Lys(Me/Et)-OH hydrochloride

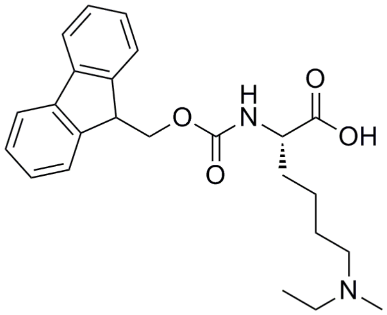

Fmoc-Lys(Me,Boc)-OH (1.00 g, 2.13 mmol) was dissolved in trifluoroacetic acid (10 mL) containing triisopropylsilane (0.48 mL, 1.1 eq.) and the solution was then stirred at room temperature for 1 hour. The solvent was then removed *in vacuo* and the resulting product was then redissolved in 20 mL of ethanol. Acetaldehyde (70 µL, 5 eq.) and $NaBH_3CN$ (147 mg, 1.1 eq.) were added and the mixture was left for 3 hours at room temperature. Then, 1 drop of concentrated HCl was added and the solvent was removed *in vacuo*. The product was extracted into dry acetone and the remaining precipitate was removed by filtration. Removal of solvent after reacidification with HCl *in vacuo* and subsequent trituration in diethyl ether afforded the product as a white solid (430 mg, 49 %). mp 84 °C (decomposition); $[\alpha]^{25}_{D}$ +20 (c = 0.01 g mL^{-1}, MeOH); IR ν_{max} 1676, 1449; ^1H NMR (400 MHz, DMSO-d_6) δ(ppm) 1.19 (t, *J* = 7.0 Hz, 3H, $NCH_2C\underline{\mathit{H}}_3$), 1.29-1.42 (m, 2H, γ-lys), 1.55-1.80 (m, 4H, β-lys, δ-lys), 2.70 (s, 3H, $NC\underline{H}_3$), 2.94-3.13 (m, 4H, ε-lys, $NC\underline{\mathit{H}}_2CH_3$), 3.95 (m 1H, α-lys), 4.21-4.37 (m, 3H, $OC\underline{\mathit{H}}_2CH$, $OCH_2C\underline{\mathit{H}}$), 7.34 (t, *J* = 7.5 Hz, 2H, 2×$FmocC_2\underline{H}$), 7.43 (t, *J* = 7.5 Hz, 2H, 2×$FmocC_3\underline{H}$), 7.65 (d, *J* = 7.5 Hz, 1H, $N\underline{\mathit{H}}CHCOOH$), 7.73 (app t, *J* = 6.5 Hz, 2H, 2×$FmocC_1\underline{H}$), 7.91 (d, *J* = 7.5 Hz, 2H, 2×$FmocC_4\underline{H}$), 10.33 (br s, 1H, $CH_2N\underline{\mathit{H}}(CH_3)(CH_2CH_3)$; ^{13}C NMR (175 MHz, DMSO-d_6) δ(ppm) 9.4 ($NCH_2\underline{\mathit{C}}H_3$), 23.2 ($\underline{\gamma}$-lys), 23.5 (δ-lys), 30.7 (β-lys), 39.0 ($N\underline{C}H_3$), 47.1 ($OCH_2\underline{C}H$), 50.5 ($N\underline{C}H_2CH_3$), 54.0 (α-Lys), 54.6 (ε-lys), 66.1 ($O\underline{C}H_2CH$), 120.6 ($Fmoc\underline{C}_4H$), 125.7 ($Fmoc\underline{C}_1H$), 127.6 ($Fmoc\underline{C}_2H$), 128.1 ($Fmoc\underline{C}_3H$), 141.2 ($Ar\underline{C}$), 144.3 ($Ar\underline{C}$), 156.7 ($NH\underline{C}OO$), 174.3 ($\underline{C}OOH$), HMBC correlation observed between the $NC\underline{\mathit{H}}_2CH_3$ protons and the ε-lysyl carbon; HRMS (ESI+) m/z calcd for $C_{24}H_{31}N_2O_4$ $(M+H)^+$: 411.2278. Found 411.2275.

b) Fmoc-Lys(Et_2)-OH hydrochloride

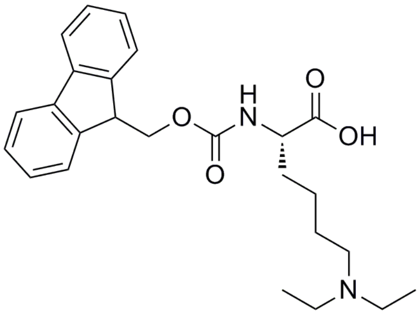

Fmoc-Lys-OH.HCl (500 mg, 1.23 mmol) and acetaldehyde (0.14 mL, 5 eq.) were mixed in 20 mL of ethanol, then $NaBH_3CN$ (163 mg, 2.1 eq.) was added portion-wise. The reaction was then left for 2 hours, after-which 1 drop of concentrated HCl was added to the mixture and the solvent was removed *in vacuo*. The product was extracted into dry acetone and the remaining precipitate was removed by filtration. Removal of solvent after reacidification with HCl *in vacuo* and subsequent trituration in diethyl ether afforded the product (as a hydrochloride) as a white solid (470 mg, 83 %). mp 106 °C (decomposition); $[\alpha]^{25}_{D}$ +30 (c = 0.01 g mL^{-1}, MeOH); IR ν_{max} 1709, 1448; ^{1}H NMR (400 MHz, DMSO-d_6) δ(ppm) 1.19 (t, *J* = 7.0 Hz, 6H, $NCH_2C\underline{H_3}$), 1.30-1.40 (m, 2H, γ-lys), 1.54-1.81 (m, 4H, β-lys, δ-lys), 2.91-3.00 (m, 2H, ε-lys), 3.00-3.12 (m, 4H, $NC\underline{H_2}CH_3$), 3.94 (m, 1H, α-lys), 4.18-4.32 (m, 3H, $OC\underline{H_2}CH$, $OCH_2C\underline{H}$), 7.33 (t, *J* = 7.5 Hz, 2H, 2×Fmoc$C_2\underline{H}$), 7.42 (t, *J* = 7.5 Hz, 2H, 2×Fmoc$C_3\underline{H}$), 7.66 (d, *J* = 8.0 Hz, 1H, N$\underline{H}$CHCOOH), 7.73 (dd, J_1 = 3.0 Hz, J_2 = 7.5 Hz, 2H, 2×Fmoc$C_1\underline{H}$), 7.90 (d, *J* = 7.5 Hz, 2H, 2×Fmoc$C_4\underline{H}$), 10.22 (br s, 1H, $CH_2N\underline{H}(CH_2CH_3)_2$); ^{13}C NMR (101 MHz, DMSO-d_6) δ(ppm) 9.2 ($N(CH_2\underline{C}H_3)_2$), 22.6 (δ-lys), 23.3 (γ-lys), 31.1 (β-lys), 46.8 ($N(\underline{C}H_2CH_3)_2$), 47.5 ($OCH_2\underline{C}H$), 51.1 (ε-lys), 54.5 (α-Lys), 66.4 ($O\underline{C}H_2CH$), 121.0 (Fmoc$\underline{C_4}$H), 126.1 (Fmoc$\underline{C_1}$H), 128.0 (Fmoc$\underline{C_2}$H), 128.6 (Fmoc$\underline{C_3}$H), 141.6 (Ar$\underline{C}$), 144.7 (Ar$\underline{C}$), 157.1 (NH$\underline{C}$OO), 174.7 ($\underline{C}$OOH), HMBC correlation observed between the $N(C\underline{H_2}CH_3)$ protons and the ε-lysyl carbon; HRMS (ESI+) m/z calcd for $C_{25}H_{33}N_2O_4$ $(M+H)^+$: 425.2435. Found 425.2436.

c) Fmoc-Lys(iPr)-OH hydrochloride

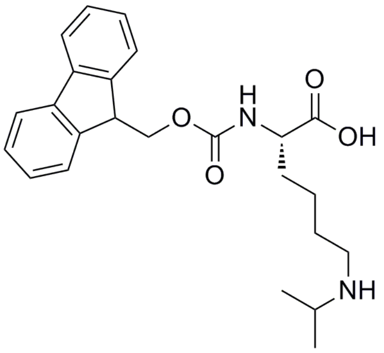

Fmoc-Lys-OH.HCl (1.00 g, 2.46 mmol) was dissolved in 20 mL of ethanol, before 1 mL of acetone was added. $NaBH_3CN$ (170 mg, 2.2 eq.) was then added portion-wise to the mixture and the reaction was left at room temperature. After 2 hours, 1 drop of concentrated HCl was added to the mixture and the solvent was removed *in vacuo*. The product was then extracted into dry acetone and the remaining precipitate was removed by filtration. Removal of solvent after reacidification with HCl *in vacuo* and subsequent trituration in diethyl ether afforded the product (as a hydrochloride) as a white solid (900 mg, 82 %). mp 95 °C (decomposition); $[\alpha]^{25}_{D}$ +34 (c = 0.01 g mL^{-1}, MeOH); IR ν_{max} 1693, 1449; ^1H NMR (400 MHz, DMSO-d_6) δ(ppm) 1.22 (d, *J* = 6.5 Hz, 6H, $NCH(C\underline{H_3})_2$), 1.31-1.44 (m, 2H, γ-lys), 1.54-1.78 (m, 4H, β-lys, δ-lys), 2.77-2.88 (m, 2H, ε-lys), 3.23 (m, 1H, $NC\underline{H}(CH_3)_2$), 3.94 (m, 1H, α-lys), 4.18-4.32 (m, 3H, $OC\underline{H_2}CH$, $OCH_2C\underline{H}$), 7.33 (t, *J* = 7.5 Hz, 2H, 2×Fmoc$C_2\underline{H}$), 7.42 (t, *J* = 7.5 Hz, 2H, 2×Fmoc$C_3\underline{H}$), 7.67 (d, *J* = 8.0 Hz, 1H, $N\underline{H}CHCOOH$), 7.73 (dd, J_1 = 3.0 Hz, J_2 = 7.5 Hz, 2H, 2×Fmoc$C_1\underline{H}$), 7.89 (d, *J* = 7.5 Hz, 2H, 2×Fmoc$C_4\underline{H}$), 8.79 (br s, 2H, $CH_2N\underline{H}CH(CH_3)_2$); ^{13}C NMR (101 MHz, DMSO-d_6) δ(ppm) 19.4 ($NCH(\underline{C}H_3)_2$), 23.7 (γ-lys), 26.1 (δ-lys), 31.1 (β-lys), 44.4 (ε-lys), 47.5 ($OCH_2\underline{C}H$), 50.1 ($N\underline{C}H(CH_3)_2$), 54.5 (α-lys), 66.5 ($O\underline{C}H_2CH$), 121.0 (Fmoc$\underline{C}_4$H), 126.2 (Fmoc$\underline{C}_1$H), 128.0 (Fmoc$\underline{C}_2$H), 128.5 (Fmoc$\underline{C}_3$H), 141.5 (Ar$\underline{C}$), 144.7 (Ar$\underline{C}$), 157.1 (NH$\underline{C}$OO), 174.7 ($\underline{C}$OOH), HMBC correlation observed between the $NC\underline{H}(CH_3)_2$ protons and the ε-lysyl carbon; HRMS (ESI+) m/z calcd for $C_{24}H_{31}N_2O_4$ $(M+H)^+$: 411.2278. Found 411.2272.

d) Fmoc-Lys(Me/iPr)-OH hydrochloride

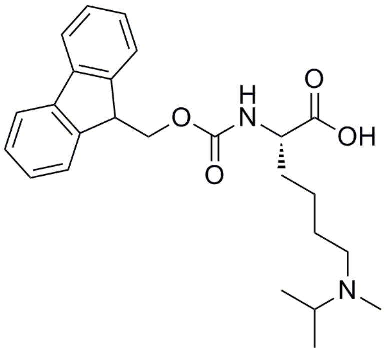

Fmoc-Lys(Me,Boc)-OH (1.00 mg, 2.13 mmol) was dissolved in trifluoroacetic acid (10 mL) and triisopropylsilane (0.48 mL, 1.1 eq.); the solution was stirred at room temperature for 1 hour. The solvent was then removed *in vacuo* and the resulting product was then redissolved in 20 mL of ethanol. Acetaldehyde (0.07 mL, 5 eq.) and $NaBH_3CN$ (147 mg, 1.1 eq.) were added and the mixture was left for 3 hours at room temperature. Then, 1 drop of concentrated HCl was added and the solvent was removed *in vacuo*. The product was extracted into dry acetone and the remaining precipitate was removed by filtration. Removal of solvent after reacidification with HCl *in vacuo* and subsequent trituration in diethyl ether afforded the product as a white solid (609 mg, 67 %). mp 90 °C (decomposition); $[\alpha]^{25}_{D}$ +22 (c = 0.01 g mL^{-1}, MeOH); IR ν_{max} 1681, 1602; ^{1}H NMR (400 MHz, DMSO-d_6) δ(ppm) 1.17-1.27 (m, 6H, NCH(C*H_3*)$_2$), 1.29-1.43 (m, 2H, γ-lys), 1.55-1.80 (m, 4H, β-lys, δ-lys), 2.58 (s, 3H, NCH_3), 2.78-3.07 (m, 3H, ε-lys, NC*H*(CH_3)$_2$), 3.94 (m, 1H, α-lys), 4.17-4.36 (m, 3H, OC*H_2*CH, OCH_2C*H*), 7.33 (t, *J* = 7.5 Hz, 2H, 2×FmocC_2H), 7.42 (t, *J* = 7.5 Hz, 2H, 2×FmocC_3H), 7.67 (d, *J* = 7.5 Hz, 1H, N*H*CHCOOH), 7.70-7.76 (m, 2H, 2×FmocC_1H), 7.90 (d, *J* = 7.5 Hz, 2H, 2×FmocC_4H); ^{13}C NMR (125 MHz, DMSO-d_6) δ(ppm) 17.1 (NCH(*C*H_3)$_2$), 23.2 (γ-lys), 23.9 (δ-lys), 30.7 (β-lys), 35.0 (N*C*H_3), 47.2 (OCH_2*C*H), 52.3 (ε-lys), 54.2 (N*C*H(CH_3)$_2$), 56.4 (α-Lys), 66.1 (O*C*H_2CH), 120.6 (FmocC_4H), 125.8 (FmocC_1H), 127.5 (FmocC_2H), 128.2 (FmocC_3H), 141.2 (ArC), 144.3 (ArC), 156.7 (NHCOO), 174.3 (COOH), HMBC correlation observed between the NC*H*(CH_3)$_2$ protons and the ε-lysyl carbon; HRMS (ESI+) m/z calcd for $C_{24}H_{31}N_2O_4$ $(M+H)^+$: 425.2435. Found 425.2432.

e) Fmoc-Orn(Me_2)-OH hydrochloride

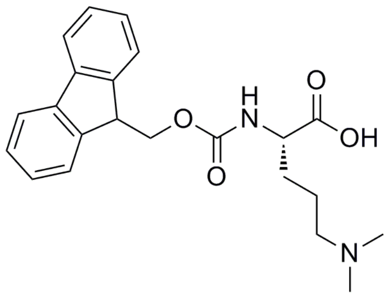

0.5 mL of aqueous HCHO (37 % in H_2O, 6 eq.) was added to a ethanolic solution of Fmoc-Orn-OH.HCl (500 mg, 1.28 mmol). $NaBH_3CN$ (170 mg, 2.2 eq.) was then added portion-wise to the mixture and the reaction was stirred at room temperature for 1 hour. After this time, 1 drop of concentrated HCl was added and the solvent was removed *in vacuo*. Removal of solvent after reacidification with HCl *in vacuo* and subsequent trituration in diethyl ether afforded the product as the hydrochloride salt (as a yellow-white solid,150 mg, 28 %). mp 145 °C (decomposition); $[\alpha]^{25}_{D}$ +30 (c = 0.01 g mL^{-1}, MeOH); IR ν_{max} 1699, 1448; ^{1}H NMR (400 MHz, DMSO-d_6) δ(ppm) 1.50-1.80 (m, 4H, $C\underline{H_2}CH_2CH_2N$, $CH_2C\underline{H_2}CH_2N$), 2.71 (d, *J* = 4.5 Hz, 6H, $N(C\underline{H}_3)_2$), 2.95-3.06 (m, 2H, $CH_2CH_2C\underline{H_2}N$), 3.97 (m, 1H, $NHC\underline{H}COOH$), 4.18-4.36 (m, 3H, $OC\underline{H_2}CH$, $OCH_2C\underline{H}$), 7.34 (t, *J* = 7.5 Hz, 2H, 2×$FmocC_2\underline{H}$), 7.42 (t, *J* = 7.5 Hz, 2H, 2×$FmocC_3\underline{H}$), 7.68-7.76 (m, 3H, 2×$FmocC_1\underline{H}$, $N\underline{H}CHCOOH$), 7.90 (d, *J* = 7.5 Hz, 2H, 2×$FmocC_4\underline{H}$), 10.20 (m, 1H, $CH_2N\underline{H}(CH_3)_2$); ^{13}C NMR (101 MHz, DMSO-d_6) δ(ppm) 21.1 ($CH_2\underline{C}H_2CH_2N$), 28.2 ($\underline{C}H_2CH_2CH_2N$), 42.3 ($N(\underline{C}H_3)_2$), 46.7 ($OCH_2\underline{C}H$), 53.6 ($NH\underline{C}HCOOH$), 56.4 ($CH_2CH_2\underline{C}H_2N$), 65.9 ($O\underline{C}H_2CH$), 120.4 ($Fmoc\underline{C_4}H$), 125.6 ($Fmoc\underline{C_1}H$), 127.4 ($Fmoc\underline{C_2}H$), 127.9 ($Fmoc\underline{C_3}H$), 141.6 ($Ar\underline{C}$), 144.7 ($Ar\underline{C}$), 157.1 ($NH\underline{C}OO$), 175.1 ($\underline{C}OOH$), HMBC correlation observed between the $N(C\underline{H}_3)_2$ protons and the $CH_2CH_2\underline{C}H_2N$ carbon; HRMS (ESI+) m/z calcd for $C_{22}H_{27}N_2O_4$ $(M+H)^+$: 383.1965. Found 3863.1959.

f) Fmoc-Orn(Et_2)-OH hydrochloride

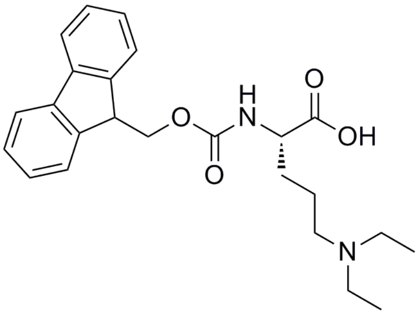

Fmoc-Orn-OH.HCl (500 mg, 1.28 mmol) was dissolved in 20 mL of ethanol, before acetaldehyde (0.36 mL, 5 eq.) was added to the solution. $NaBH_3CN$ (170 mg, 2.2 eq.) was then added portion-wise to the mixture and the reaction was stirred at room temperature for 3 hours. 1 drop of concentrated HCl was thenadded and the solvent was removed *in vacuo*. The product was then extracted into dry acetone and the remaining precipitate was removed by filtration. Removal of solvent after reacidification in HCl *in vacuo* and subsequent trituration in diethyl ether afforded the product (as the hydrochloride) as a white solid (450 mg, 79 %). mp 180 °C (decomposition); $[\alpha]^{25}_{D}$ +43 (c = 0.01 g mL^{-1}, MeOH); IR ν_{max} 1744, 1444; ^{1}H NMR (400 MHz, DMSO-d_6) δ(ppm) 1.19 (t, J = 7.5 Hz, 6H, $N(CH_2C\underline{H_3})_2$), 1.59-1.84 (m, 4H, $C\underline{H_2}CH_2CH_2N$, $CH_2C\underline{H_2}CH_2N$), 2.91-3.10 (m, 6H, $CH_2CH_2C\underline{H_2}N$, $N(C\underline{H_2}CH_3)_2$), 3.99 (m, 1H, $NHC\underline{H}COOH$), 4.18-4.36 (m, 3H, $OC\underline{H_2}CH$, $OCH_2C\underline{H}$), 7.33 (t, J = 7.5 Hz, 2H, 2×Fmoc$C_2\underline{H}$), 7.42 (t, J = 7.5 Hz, 2H, 2× Fmoc$C_3\underline{H}$), 7.73 (app t, J = 7.5 Hz, 3H, 2× Fmoc$C_1\underline{H}$, $N\underline{H}CHCOOH$), 7.89 (d, J = 7.5 Hz, 2H, 2× Fmoc$C_4\underline{H}$), 10.38-10.50 (br s, 1H, $CH_2N\underline{H}(CH_2CH_3)_2$); ^{13}C NMR (101 MHz, DMSO-d_6) δ(ppm) 8.5 ($N(CH_2\underline{C}H_3)_2$), 20.1 ($CH_2\underline{C}H_2CH_2N$), 28.2 ($\underline{C}H_2CH_2CH_2N$), 46.3 ($N(\underline{C}H_2CH_3)_2$), 46.8 ($OCH_2\underline{C}H$), 50.1 ($CH_2CH_2\underline{C}H_2N$), 53.4 ($NH\underline{C}HCOOH$), 65.9 ($O\underline{C}H_2CH$), 120.4 (Fmoc$\underline{C_4}$H), 125.5 (Fmoc$\underline{C_1}$H), 127.4 (Fmoc$\underline{C_2}$H), 127.8 (Fmoc$\underline{C_3}$H), 141.6 (Ar$\underline{C}$), 144.7 (Ar$\underline{C}$), 157.1 ($NH\underline{C}OO$), 175.1 ($\underline{C}OOH$), HMBC correlation observed between the $N(C\underline{H_2}CH_3)_2$ protons and the $CH_2CH_2\underline{C}H_2N$ carbon; HRMS (ESI+) m/z calcd for $C_{24}H_{31}N_2O_4$ $(M+H)^+$: 411.2278. Found 411.2276.

g) Fmoc-Lys(Cr)-OH

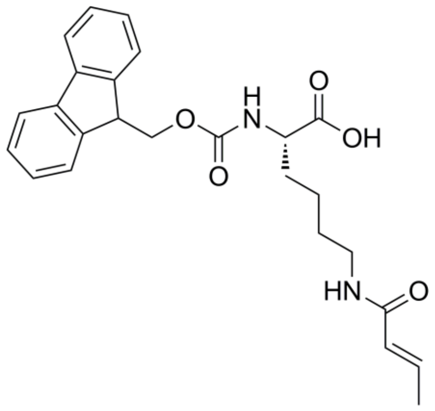

Fmoc-Lys-OH (5.00 g, 13.6 mmol) was dissolved in 10 mL of DMF and subsequently diluted with 100 mL of MeCN. K_2CO_3 (3.75 g, 27.1 mmol, 2.0 eq.) and crotonic anhydride (2.20 mL, 14.0 mmol, 1.1 eq.) were added and the solution stirred at room temperature overnight. The solvent was removed *in vacuo*, the solid resuspended in 1M HCl solution and extracted with CH_2Cl_2 (2 × 300 mL). The organic layers were combined and the solvent was removed *in vacuo* to yield the product (~90 % purity) as a white solid (5.94 g, 96 %). For characterisation purposes, the product was purified by column chromatography (0 – 10 % MeOH in CH_2Cl_2, 0.2 % AcOH). mp 79 °C (decomposition); $[\alpha]^{25}_D$ +1 (c = 0.01 g mL^{-1}, MeOH); IR ν_{max} 1699, 1635, 1540; ^{1}H NMR (400 MHz, $CDCL_3$) δ(ppm) 1.29-1.61 (m, 4H, γ-lys, δ-lys), 1.70-1.82 (m, 4H, β-lys, NHCOCHCHC$\underline{H}_3$), 1.84-1.93 (m, 1H, β-lys), 3.22-3.34 (m, 2H, ε-lys), 4.20 (t, *J* = 7.0 Hz, $OCH_2C\underline{H}$), 4.30-4.42 (m, 4H, α-lys, $OC\underline{H}_2CH$), 5.76 (d, *J* = 15.0 Hz, 1H, NHCOC$\underline{H}$CHCH_3), 5.89 (d, J = 8.0 Hz, 1H, N$\underline{H}$CHCOOH), 6.11 (t, J = 5.5 Hz, 1H, N$\underline{H}$COCHCHCH_3), 6.82 (app dt, J_1 = 15.0 Hz, J_2 = 7.0 Hz, 1H, NHCOCHC$\underline{H}$CH_3), 7.27 (t, *J* = 7.5 Hz, 2H, 2×Fmoc$C_2\underline{H}$), 7.37 (t, *J* = 7.5 Hz, 2H, 2×Fmoc$C_3\underline{H}$), 7.50-7.62 (m, 2H, 2×Fmoc$C_1\underline{H}$), 7.62 (d, *J* = 7.5 Hz, 2H, 2×Fmoc$C_4\underline{H}$; ^{13}C NMR (101 MHz, CDCl3) δ(ppm) 17.7 (NHCOOCH$\underline{C}H_3$), 22.7 (γ-lys), 28.7 (δ-lys), 31.8 (β-lys), 39.1 (ε-lys), 47.0 ($OCH_2\underline{C}H$), 53.5 (α-Lys), 67.1 ($O\underline{C}H_2CH$), 119.9 (Fmoc$\underline{C}_4$H), 124.5 (NHCOCH$\underline{C}$HCH_3), 125.1 (Fmoc$\underline{C}_1$H), 127.1 (Fmoc$\underline{C}_2$H), 127.7 (Fmoc$\underline{C}_3$H), 140.6 (NHCO$\underline{C}$HCHCH_3), 141.2 (Ar$\underline{C}$), 143.6 (Ar$\underline{C}$), 156.4 (NH$\underline{C}$OO), 166.9 (NH$\underline{C}$OCHCHCH_3), 176.7 ($\underline{C}$OOH); HRMS (ESI+) m/z calcd for $C_{25}H_{28}N_2O_5Na$ $(M+Na)^+$: 459.1890. Found 459.1874.

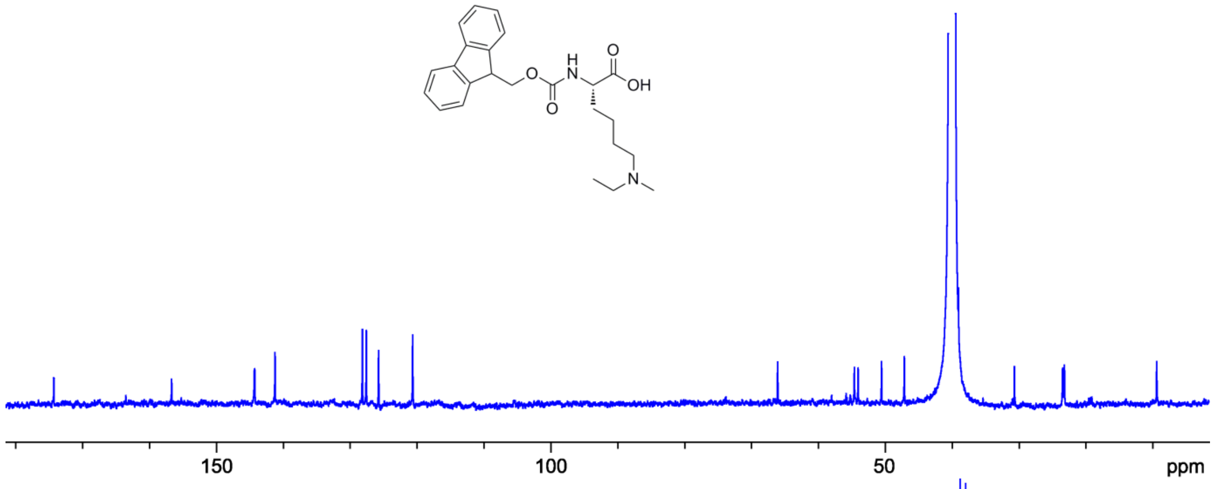
150
100
50
ppm

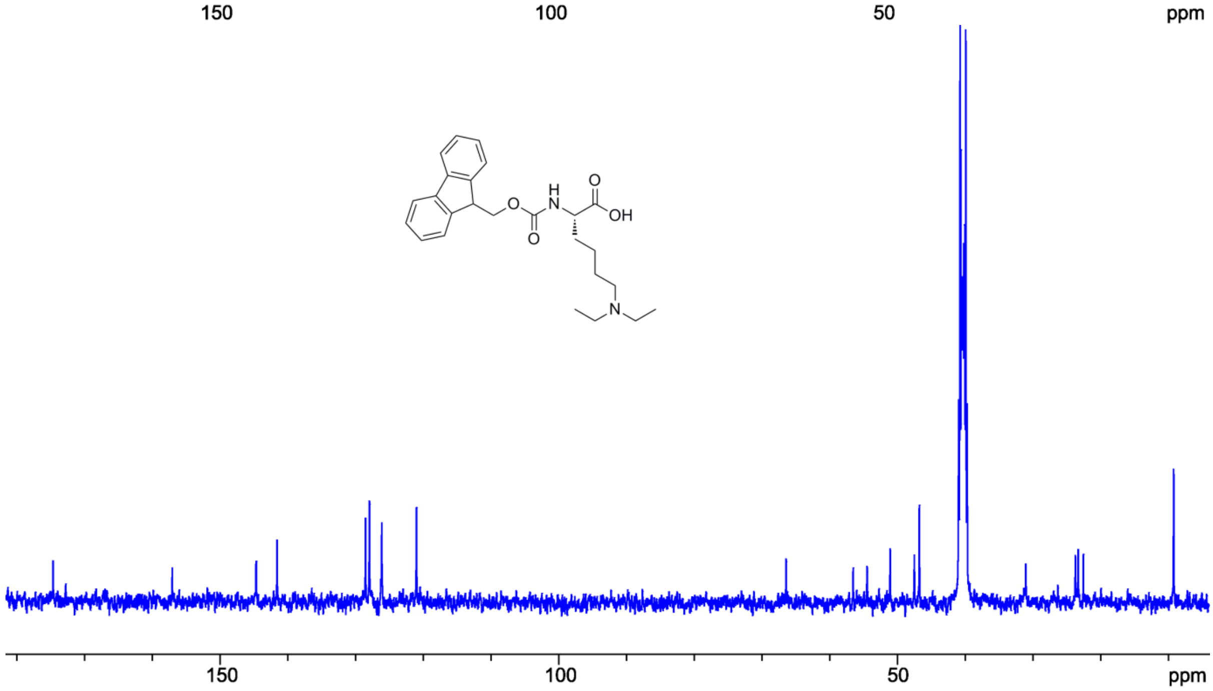
150
100
50
ppm

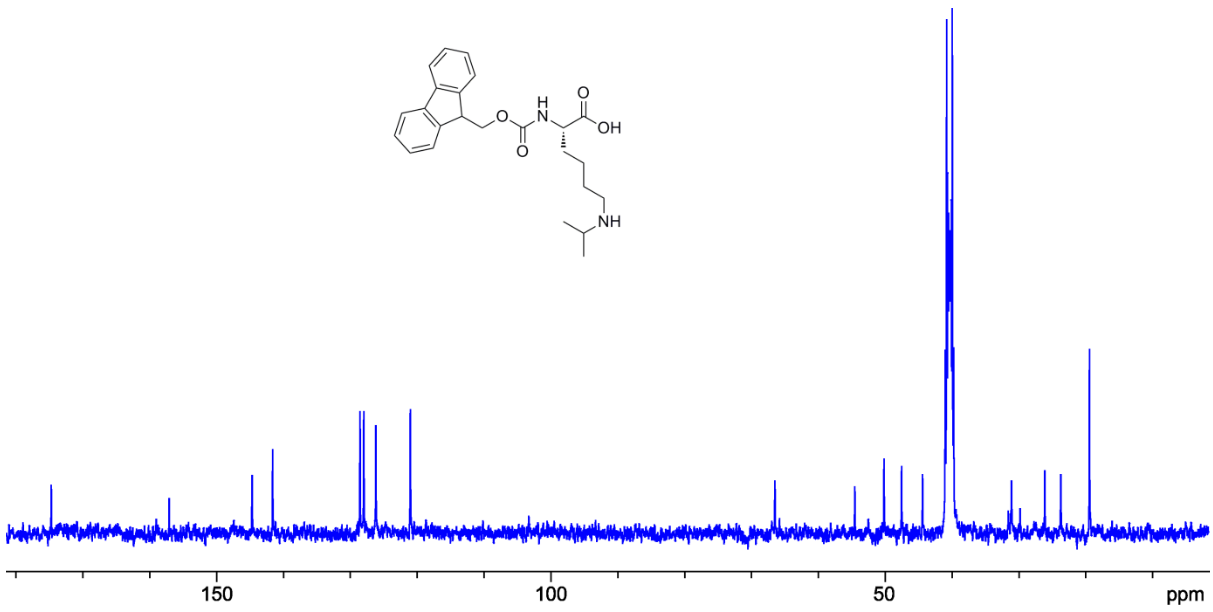
150
100
50
ppm

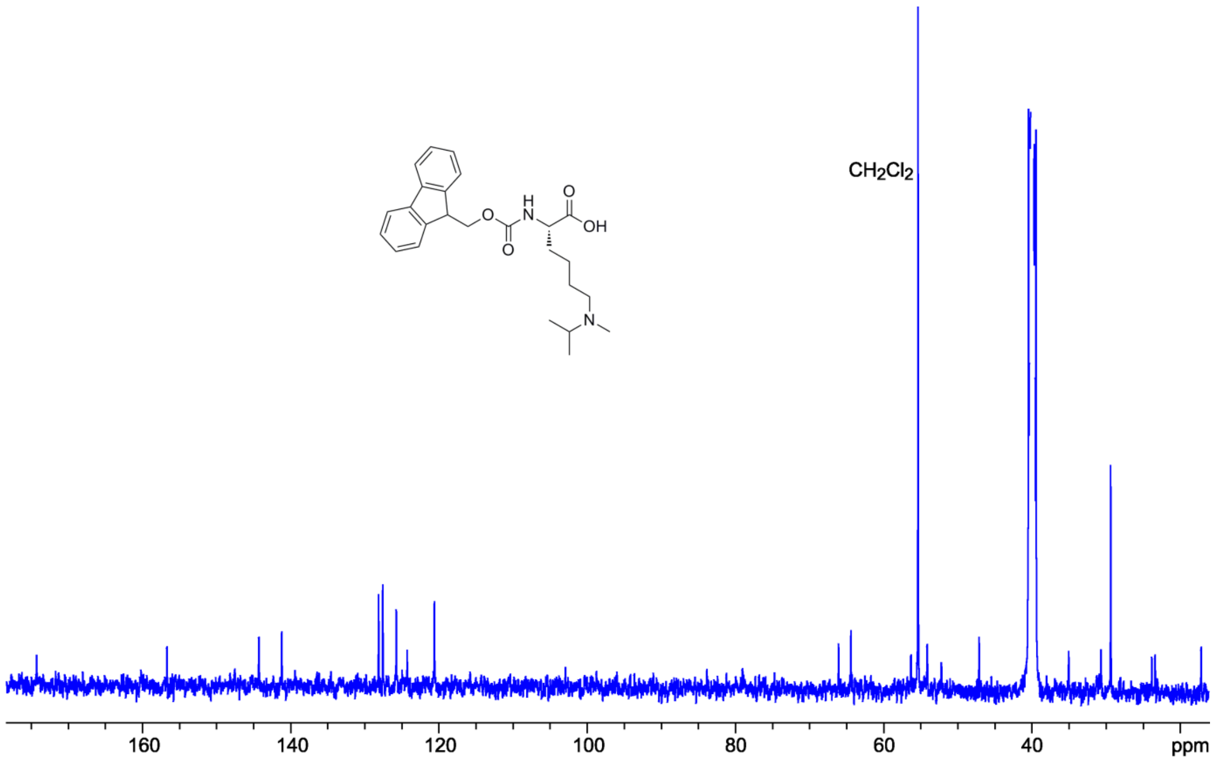
CH2Cl2
160
140
120
100
80
60
40
ppm

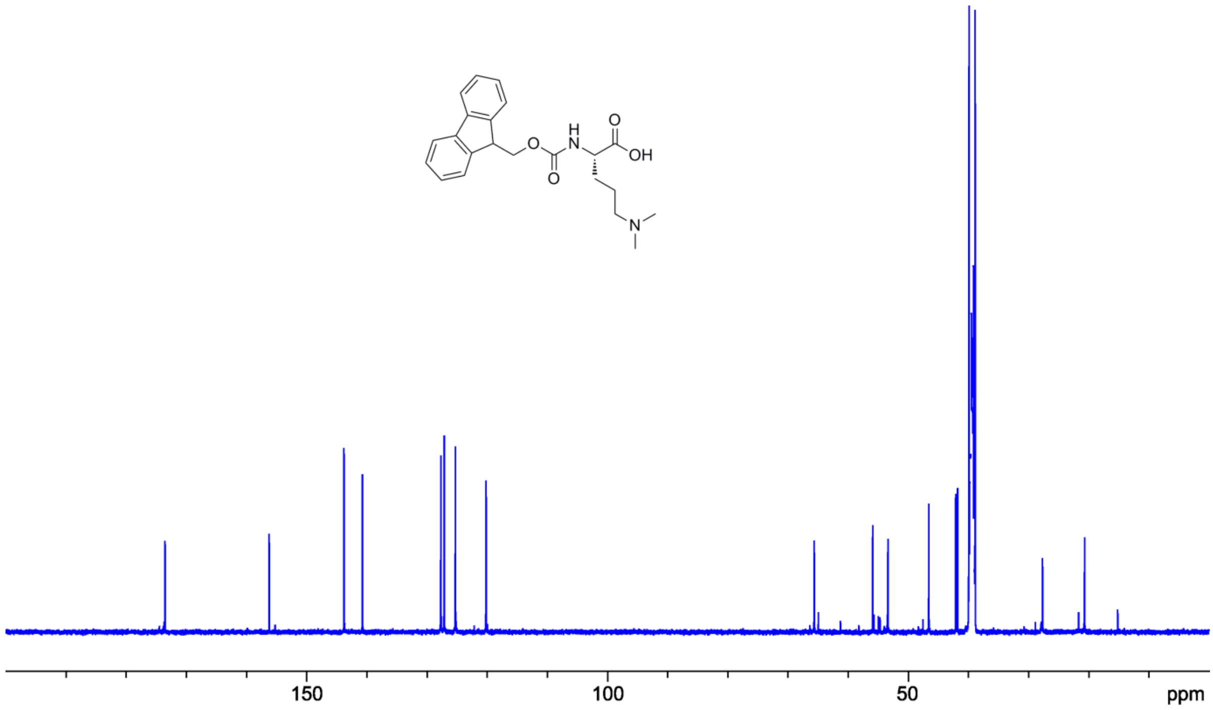
150
100
50
ppm

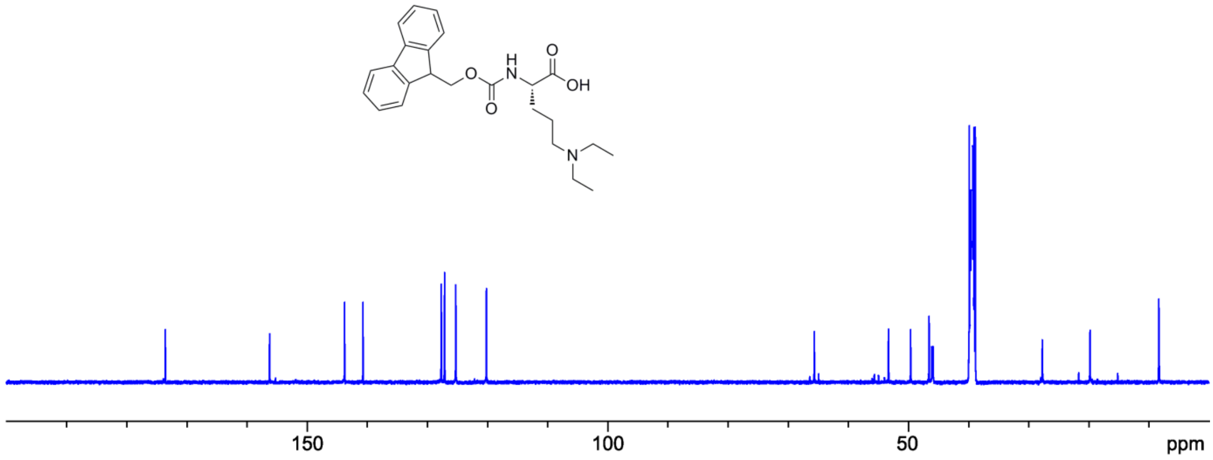
O
H
N
O
O
OH
N
150
100
50
ppm

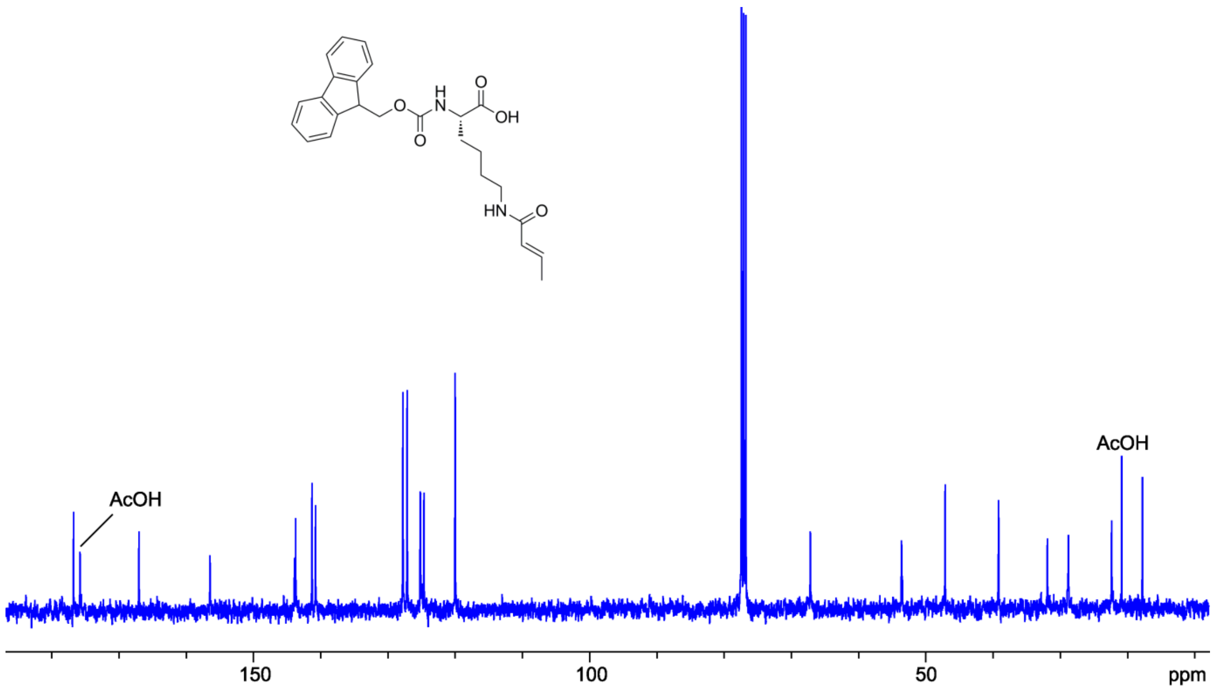
O
H
N
O
O
OH
HN
O
AcOH
AcOH
150
100
50
ppm

Peptide Synthesis

Synthesis of substrate peptides was carried out using standard Fmoc-mediated solid phase peptide synthesis using a CS336X peptide synthesiser (CSBio, USA). Peptides were synthesised on 4-methylbenzhydrylamine (MBHA) resin (Novabiochem) and cleaved using trifluoroacetic acid/triisopropylsilane (97.5 % / 2.5 % w/v) solution. Peptides were purified by LC/MS using an Agilent Technologies 2100 series purification system, equipped with an Agilent 6200 quadrupole mass spectrometer.

Activity Assays

MALDI-TOF activity assays were carried out in 20 µL final volume solutions containing enzyme (1-2 µM), peptide (10 µM), 2OG (50 µM), ascorbate (100 µM) and ammonium ferrous sulphate (10 µM), buffered in 50 mM HEPES pH 7.5. Peptide concentrations were calibrated relative to a stock solutions of Lys9(Me/Et) and Lys36(Me/Et) peptides (100 µM) using peak intensities from MALDI-TOF MS spectra. Samples were incubated for 1 hour at 25 °C before analysis by MALDI-TOF. Competition experiments were carried out with equivalent concentrations of peptides (5 µM), as assessed by MALDI-TOF peak intensities. NMR assays were carried out in deuterated ammonium formate buffer at 25 °C, as reported.[1]

Kinetic Analyses

Determination of kinetic parameters was carried out using MALDI-TOF activity. Solutions (10 µL final volume) containing JMJD2E (500 nM), peptide (20 – 510 µM), 2OG (200 µM), ascorbate (100 µM) and ammonium ferrous sulphate (10 µM), buffered in 50 mM HEPES pH 7.5, were monitored over initial time points (0-11 minutes) by MALDI-TOF. Kinetic parameters were calculated from plots of initial reaction rates versus peptide concentrations using GraphPad Prism 5.

Mass Spectrometry

MALDI-TOF MS analyses were performed using a Waters Micromass™ MALDI micro MX™ mass spectrometer in positive ion reflectron mode using α-cyano-4-hydroxycinnamic acid matrix. All MS data was analysed using Masslynx™ software.

NMR Spectroscopy

NMR analyses were performed using a Bruker AVIII 700 spectrometer equipped with an inverse TCI cryoprobe optimised for ^{1}H observation and running TOPSPIN 2 software. The solvent deuterium signal was used as an internal lock signal and the HDO signal was reduced by either presaturation or excitation sculpting where necessary. ^{1}H chemical shifts are reported relative to the HDO signal at 25 °C (δ_H 4.72 ppm).

Figures

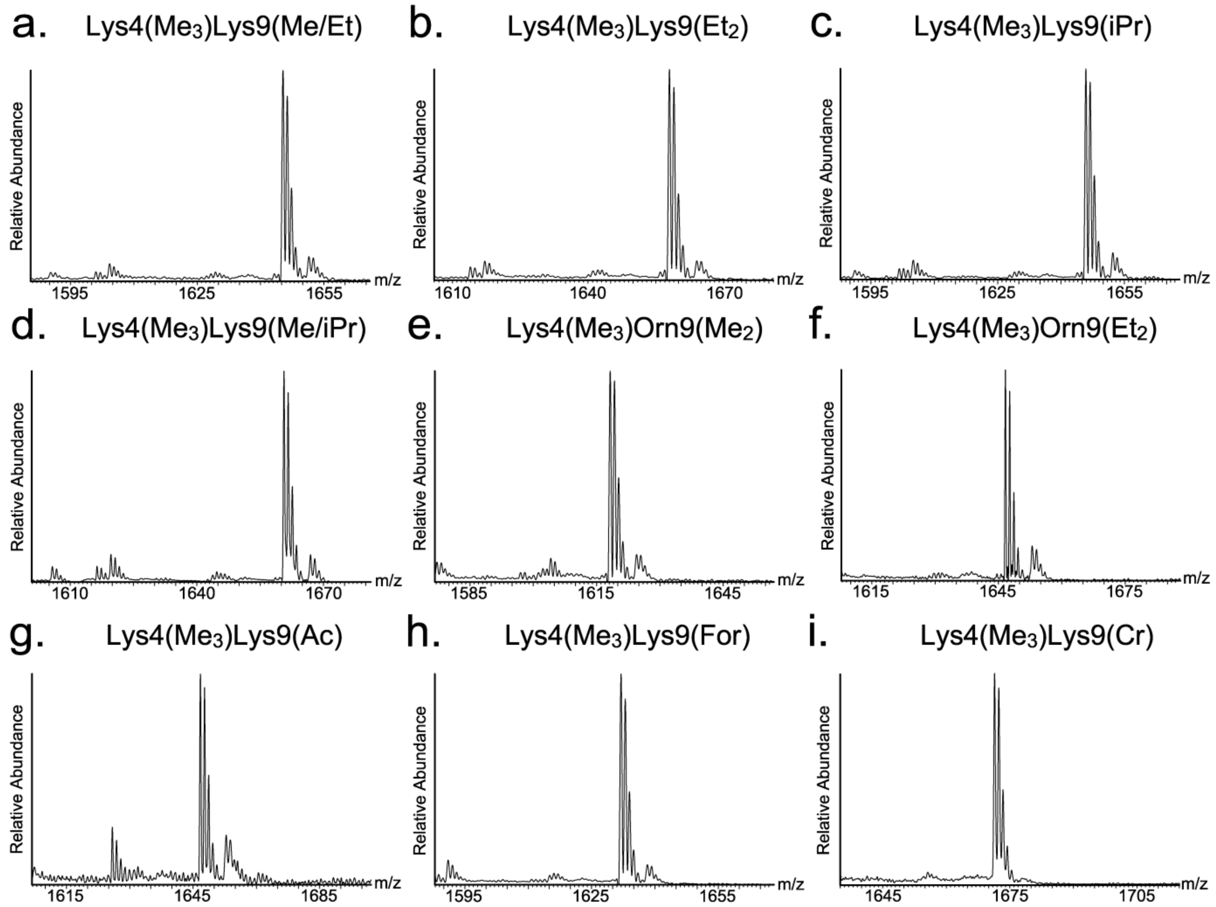

Figure S1 MS spectra of potential substrates: (a) ART-Lys(Me_3)-QTAR-Lys(Me/Et)-STGGKA, (b) ART-Lys(Me_3)-QTAR-Lys(Et_2)-STGGKA, (c) ART-Lys(Me_3)-QTAR-Lys(iPr)-STGGKA, (d) ART-Lys(Me_3)-QTAR-Lys(Me/iPr)-STGGKA, (e) ART-Lys(Me_3)-QTAR-Orn(Me_2)-STGGKA, (f) ART-Lys(Me_3)-QTAR-Orn(Et_2)-STGGKA, (g) ART-Lys(Me_3)-QTAR-Lys(Ac)-STGGKA, (h) ART-Lys(Me_3)-QTAR-Lys(For)-STGGKA, and (i) ART-Lys(Me_3)-QTAR-Lys(Cr)-STGGKA.

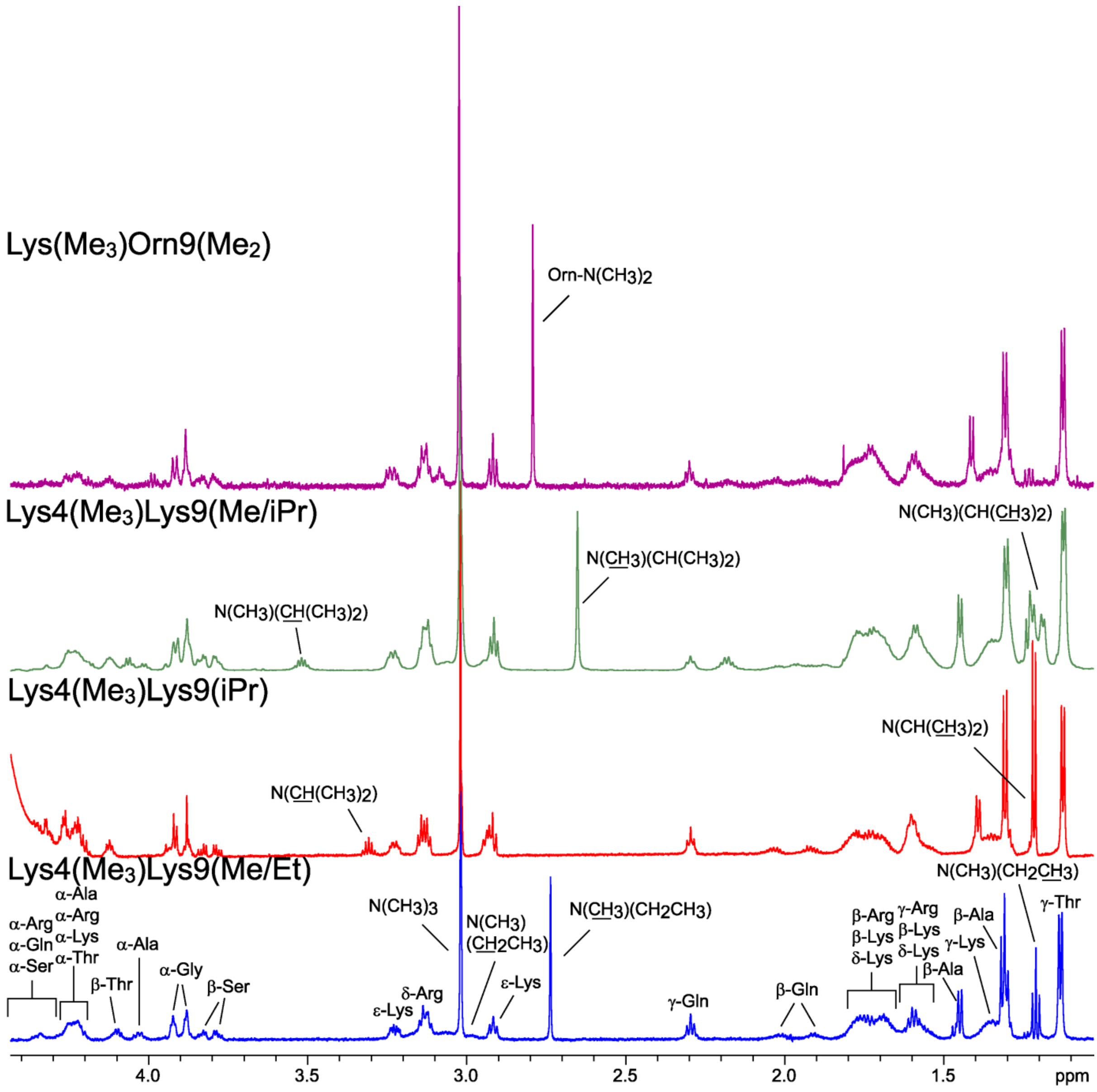

Figure S2 ^{1}H NMR spectra of potential substrates: ART-Lys(Me_3)-QTAR-Lys(Me/Et)-STGGKA (blue), ART-Lys(Me_3)-QTAR-Lys(iPr)-STGGKA (red), ART-Lys(Me_3)-QTAR-Lys(Me/iPr)-STGGKA (green) and ART-Lys(Me_3)-QTAR-Orn(Me_2)-STGGKA (purple).

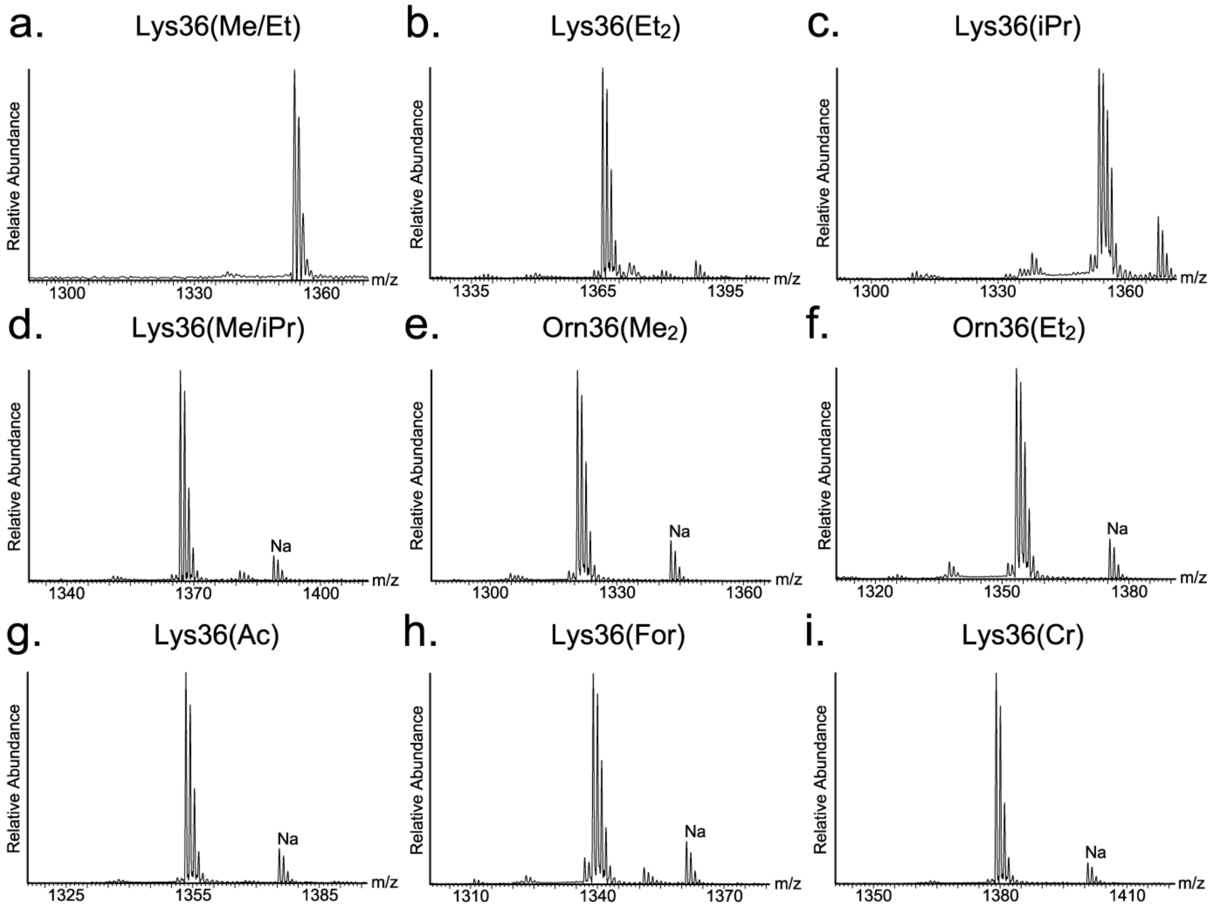

Figure S3 MS spectra of potential substrates: (a) PATGGV-Lys(Me/Et)-KPHRY, (b) PATGGV-Lys(Et_2)-KPHRY, (c) PATGGV-Lys(iPr)-KPHRY, (d) PATGGV-Lys(Me/iPr)-KPHRY, (e) PATGGV-Orn(Me_2)-KPHRY, (f) PATGGV-Orn(Et_2)-KPHRY, (g) PATGGV-Lys(Ac)-KPHRY, (h) PATGGV-Lys(For)-KPHRY, and (i) PATGGV-Lys(Cr)-KPHRY.

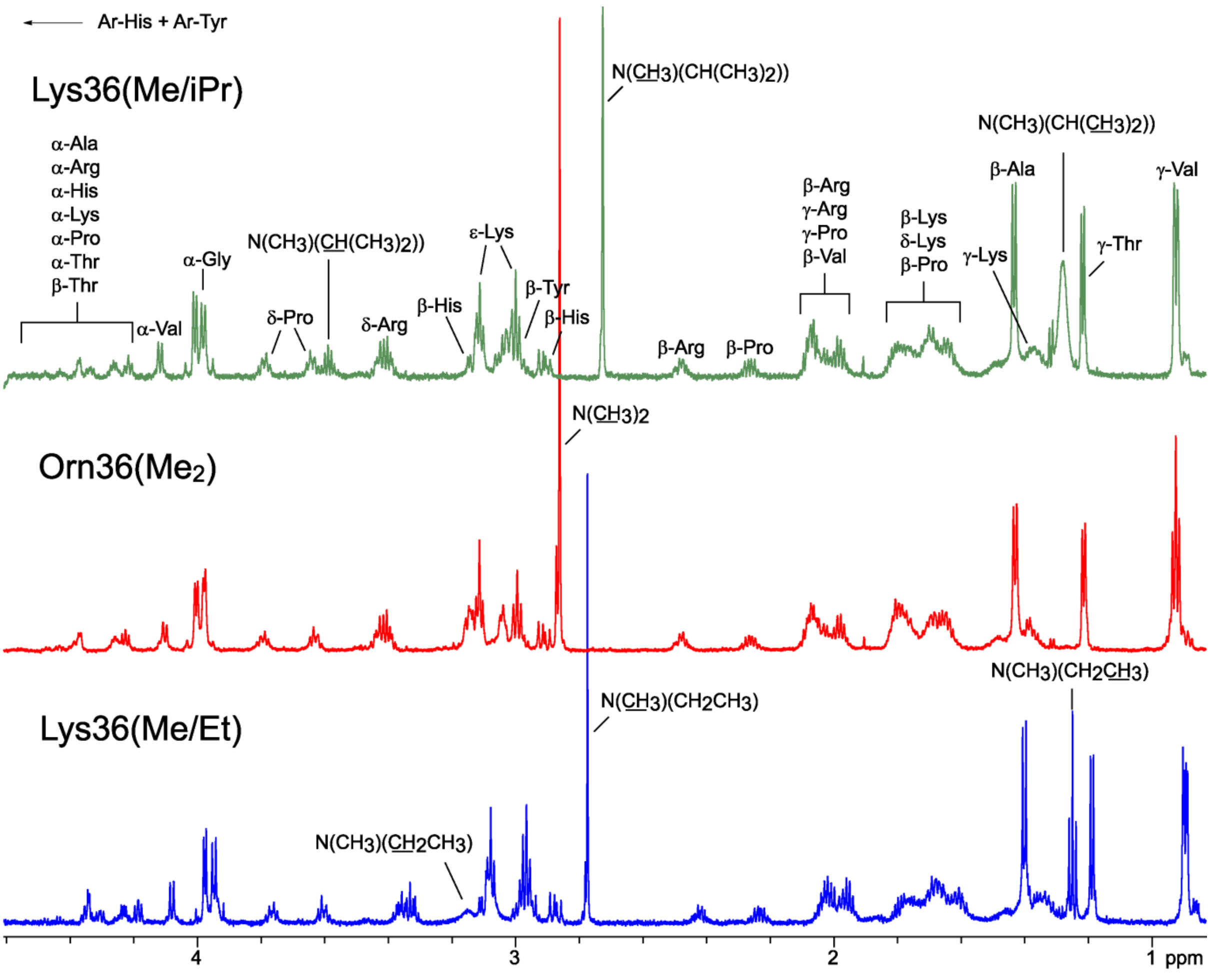

Figure S4 ^{1}H NMR spectra of potential substrates: PATGGV-Lys(Me/Et)-KPHRY (blue), PATGGV-Orn(Me_2)-KPHRY (red) and PATGGV-Lys(Me/iPr)-KPHRY (green).

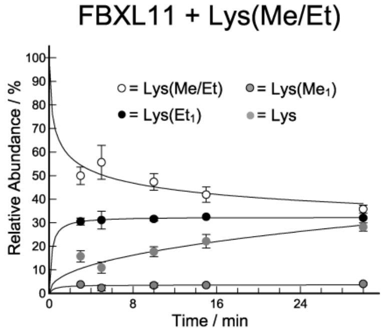

Figure S5 Relative abundances of peptides over time in samples containing H3 Lysine-36(Me/Et) peptide (sequence PATGGV-Lys(Me/Et)-KPHRY) and FBXL11 under standard conditions over 30 minutes at 25 °C (assessed by MALDI-TOF analysis). Errors are given as standard deviations of the mean (n = 3).

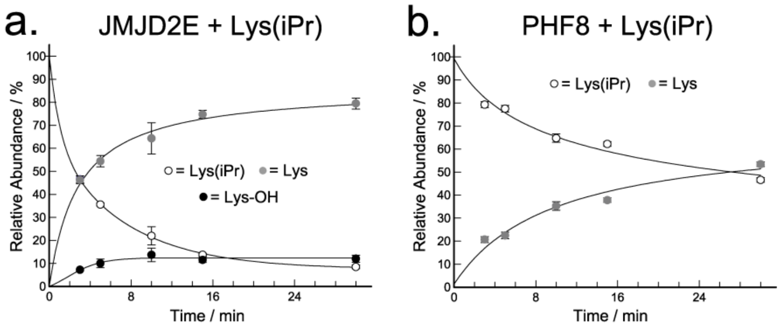

Figure S6 Analysis of the reactions of JMJD2E and PHF8 with H3 Lysine-9(iPr) peptide (assessed by MALDI-TOF analysis). (a) Relative abundances of peptides over time in samples containing H3 Lysine-9(iPr) peptide (sequence ART-Lys(Me_3)-QTAR-Lys(iPr)-STGGKA) and JMJD2E under standard conditions over 30 minutes at 25 °C. (b) Relative abundances of peptides over time in samples containing H3 Lysine-9(iPr) peptide (sequence ART-Lys(Me_3)-QTAR-Lys(iPr)-STGGKA) and PHF8 under standard conditions over 30 minutes at 25 °C. Errors are given as standard deviations of the mean (n = 3).

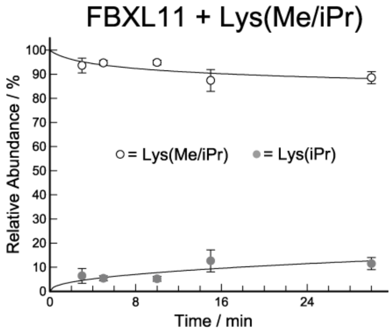

Figure S7 Relative abundances of peptides over time in samples containing H3 Lysine-36(Me/iPr) peptide (sequence PATGGV-Lys(Me/iPr)-KPHRY) and FBXL11 under standard conditions over 30 minutes at 25 °C (assessed by MALDI-TOF analysis). Errors are given as standard deviations of the mean (n = 3).

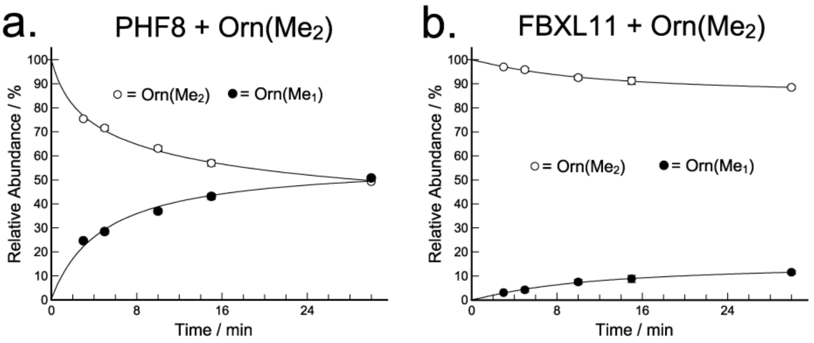

Figure S8 Analysis of the reactions of PHF8 and FBXL11 with Orn(iPr) peptides (assessed by MALDI-TOF analysis). (a) Relative abundances of peptides over time in samples containing H3 Ornithine-9(Me_2) peptide (sequence ART-Lys(Me_3)-QTAR-Orn(Me_2)-STGGKA) and PHF8 under standard conditions over 30 minutes at 25 °C. (b) Relative abundances of peptides over time in samples containing H3 Ornithine-36(Me_2) peptide (sequence PATGGV-Orn(Me_2)-KPHRY) and FBXL11 over 30 minutes at 25 °C. Errors (standard deviations of the mean, n = 3) were too small to visualise.

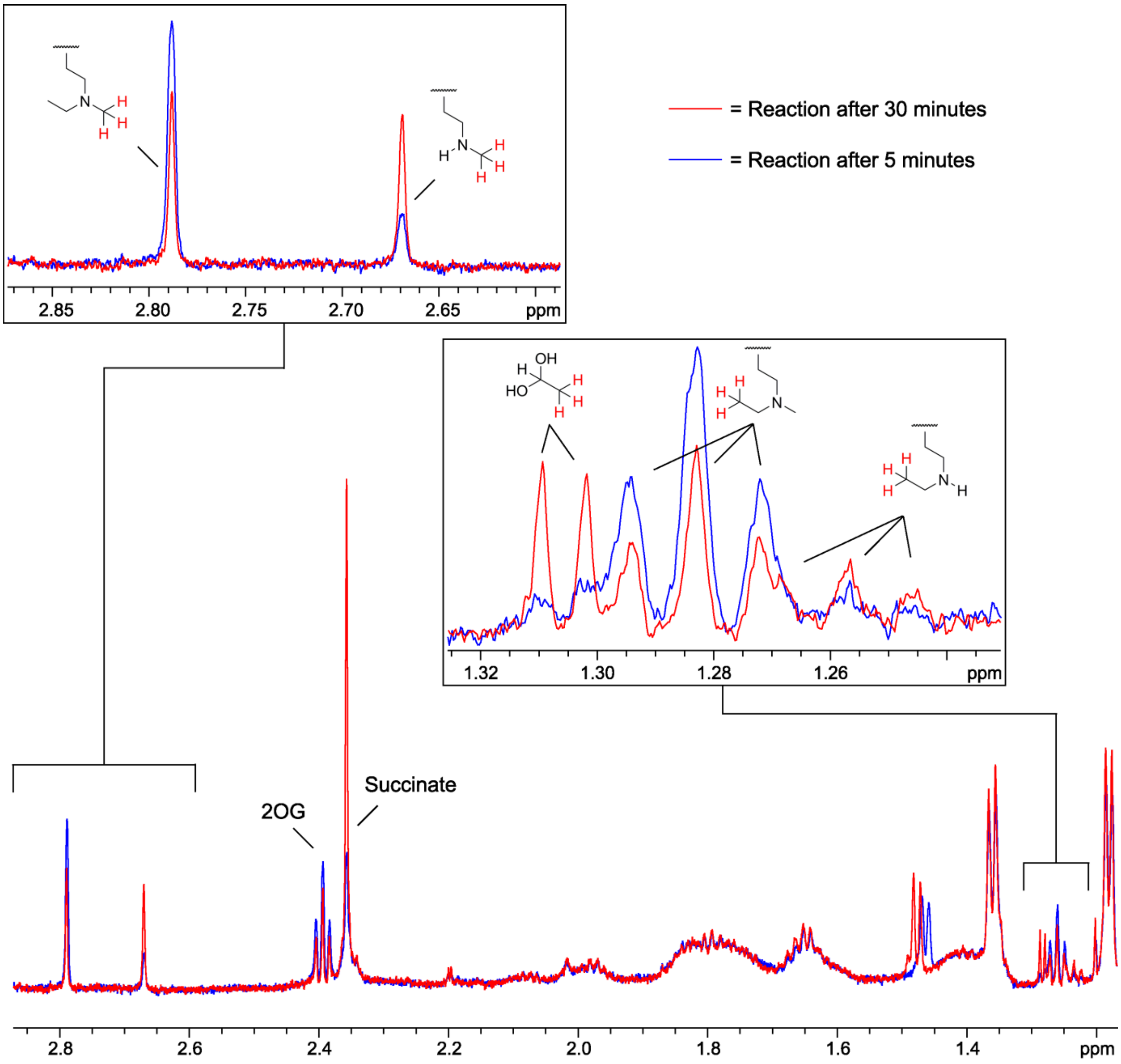

Figure S9 ^{1}H NMR spectra of a sample containing H3 Lysine-9(Me/Et) peptide (sequence ART-Lys(Me_3)-QTAR-Lys(Me/Et)-STGGKA) and JMJD2E under standard conditions after 5 and 30 minutes at 25 °C. The resonances are assigned as follows: hydrated acetaldehyde (δ_H 1.31 ppm, 3H, doublet), monoethylated product (δ_H 1.26 ppm, 3H, triplet), monomethylated product (δ_H 2.67 ppm, 3H, singlet).

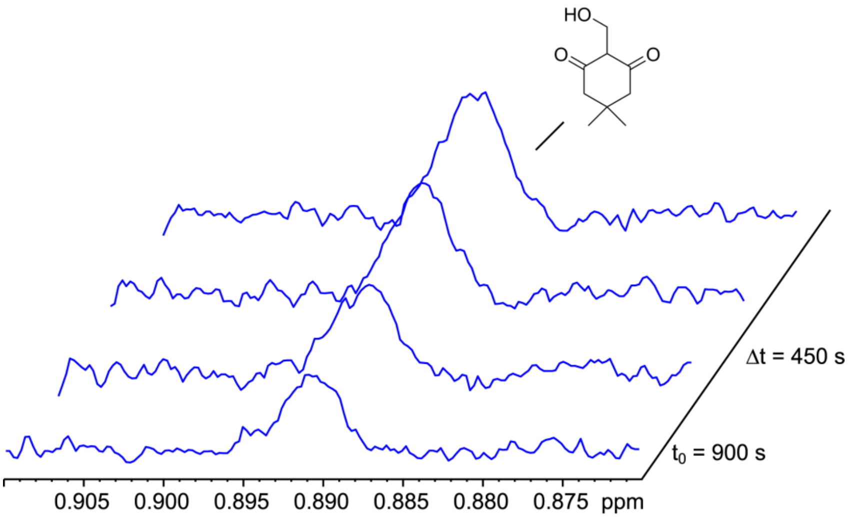

Figure S10 ^{1}H NMR spectra showing HCHO release and capture by dimedone during JMJD2E catalysed demethylation of H3 Lysine-9(Me/Et) peptide at 25 °C. The ^{1}H resonance corresponding to the methyl protons of the dimedone-HCHO adduct is highlighted (for more detailed explanation of this method, see Ref. 1 below).

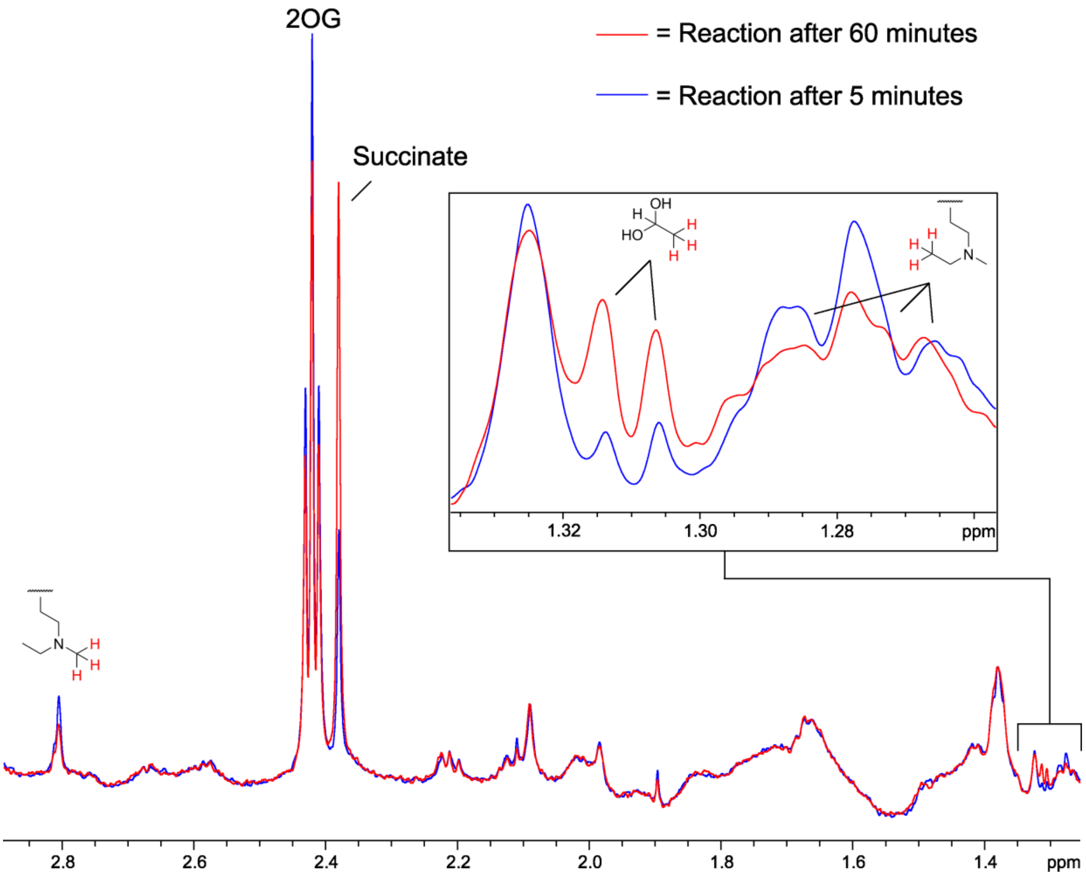

Figure S11 ^{1}H NMR spectra of a sample containing the H3 Lysine-9(Me/Et) peptide and PHF8 under standard conditions after 5 minutes (blue) and 60 minutes (red) at 25 °C. No evidence for the formation of monomethylated peptide was apparent (unlike in the NMR experiments with JMJD2E, Figure S9), suggesting that de-ethylation of the Lys(Me/Et) substrate to Lys(Me_1) peptide is not efficiently catalysed by PHF8 under the tested conditions. However, hydrated acetaldehyde was observed, indicating de-ethylation of the intermediate Lys(Et_1) peptide (formed after demethylation of Lys(Me/Et)). ^{1}H resonances corresponding to methyl and ethyl protons, as well as those corresponding to acetaldehyde are highlighted.

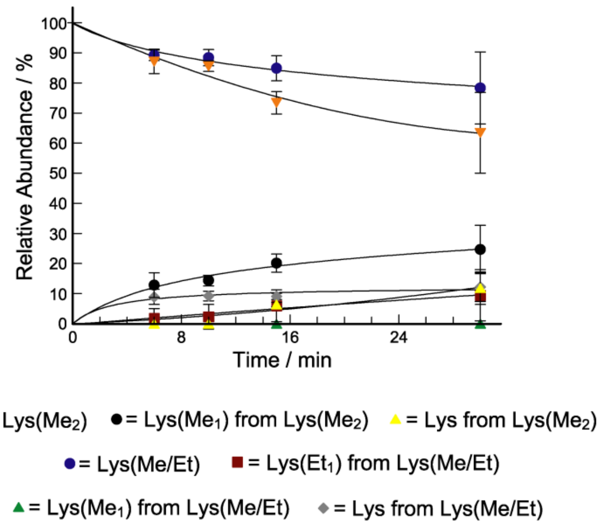

Figure S12 Relative abundances of peptides over time in samples containing H3 Lysine-9(Me/Et) peptide and H3 Lysine-9(Me_2) peptide (sequence ART-Lys(Me_3)-QTAR-Lys(Me_2)-STGGK) in a 1:1 ratio, after incubation with PHF8 at 25 °C (assessed by MALDI-TOF analysis). The monomethylated peptide derived from the Lys(Me_2) peptide is present over all time points (black circles), whereas the monomethylated peptide derived from the Lys(Me/Et) peptide is not observed. However, unalkylated peptides derived from both Lys(Me/Et) peptide (grey diamonds) and Lys(Me_2) peptide (yellow triangles) are present, suggesting that PHF8 catalysed de-alkylation of Lys(Me/Et) peptide predominantly proceeds via sequential demethylation, then de-ethylation of monoethylated peptide (see Scheme 1 in main text). Errors are given as standard deviations of the mean (n = 2).

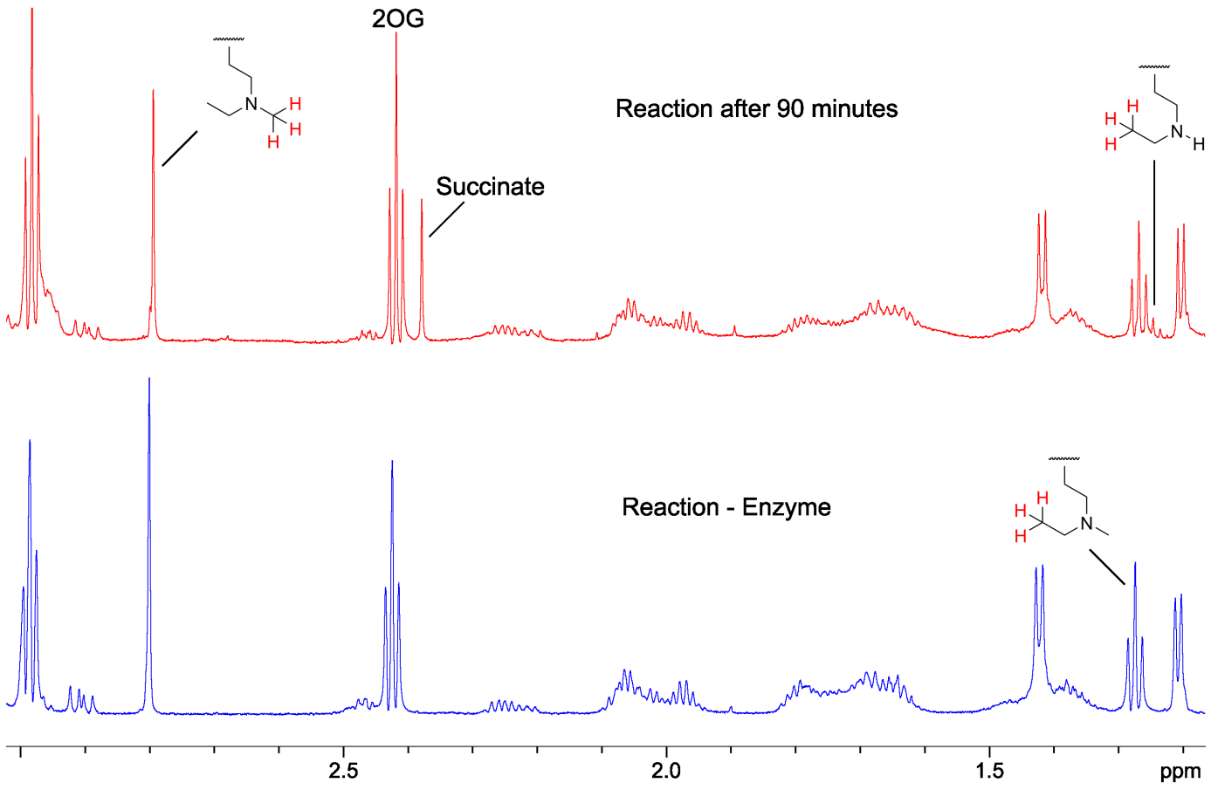

Figure S13 ^{1}H NMR spectrum of a sample containing the H3 Lysine-36(Me/Et) peptide (sequence PATGGV-Lys(Me/Et)-KPHRY) and FBXL11 under standard conditions after 90 minutes at 25 °C. A spectrum after an identical incubation but without enzyme is shown below. In the presence of FBXL11, a low level ^{1}H resonance corresponding to the terminal ethyl protons of the monoethylated peptide are observed (at δ_H 1.26 ppm); however, acetaldehyde is not formed, suggesting further de-ethylation of the monoethylated peptide does not occur under the tested conditions.

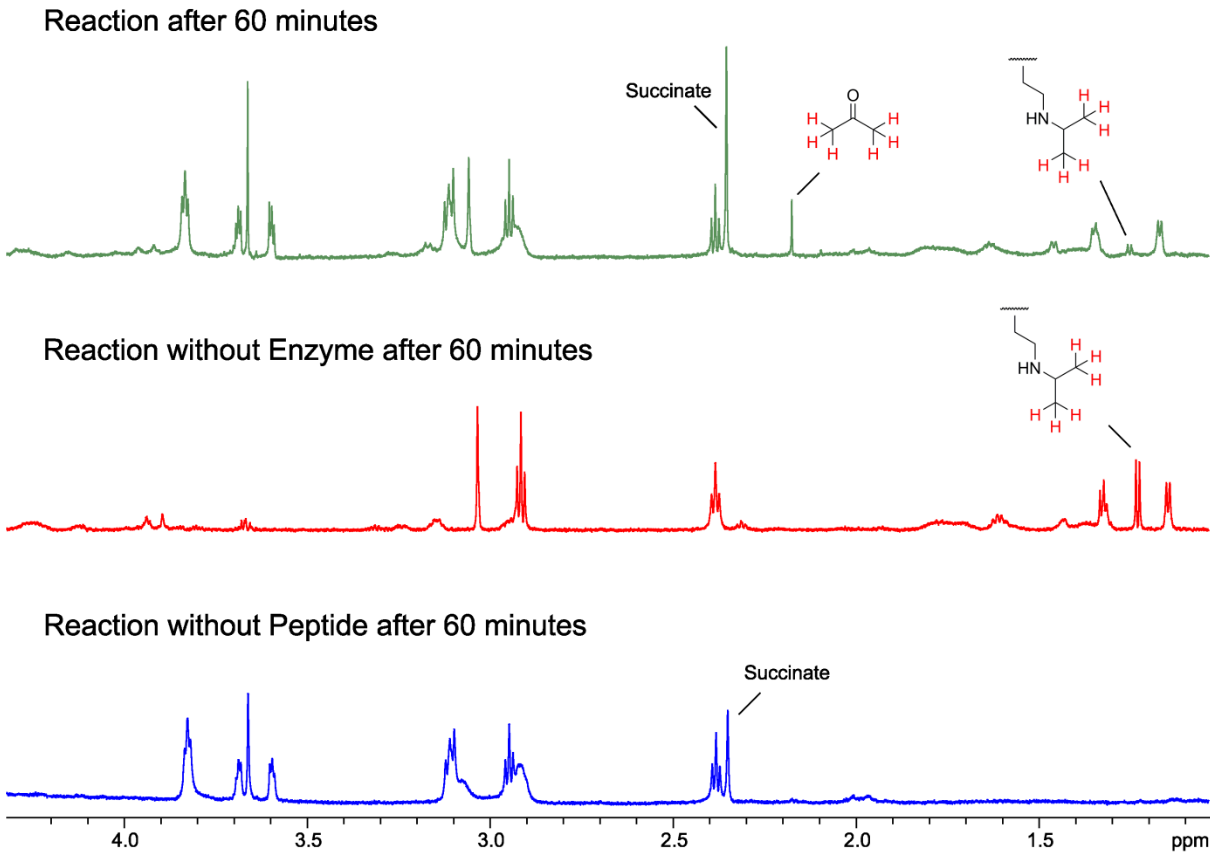

Figure S14 ^{1}H NMR spectra monitoring reactions of JMJD2E with the H3 Lysine-9(iPr) peptide. Top; ^{1}H NMR spectrum of a sample containing H3 Lysine-9(iPr) peptide (sequence ART-Lys(Me_3)-QTAR-Lys(iPr)-STGGKA) and JMJD2E under standard conditions after 60 minutes at 25 °C. De-isopropylation of the substrate peptide is implied by a decrease in the intensity of the doublet ^{1}H resonance at δ_H 1.22 ppm. A new singlet ^{1}H resononance at δ_H 2.17 ppm is observed, suggesting formation of acetone. Also, a small singlet ^{1}H resononance at δ_H 2.09 ppm is observed. Middle; ^{1}H NMR spectrum of an identical incubation but without enzyme. The singlet ^{1}H resononances at δ_H 2.17 ppm and δ_H 2.09 ppm are not observed. Bottom; ^{1}H NMR spectrum of an identical incubation but without peptide. The singlet ^{1}H resononances at δ_H 2.17 ppm and δ_H 2.09 ppm are not observed. The resonances between δ_H 2.9 ppm and δ_H 3.9 ppm (excluding the triplet 2OG resonance at δ_H 2.95 ppm) correspond to protons of ascorbate and HEPES buffer (from the enzyme stock).

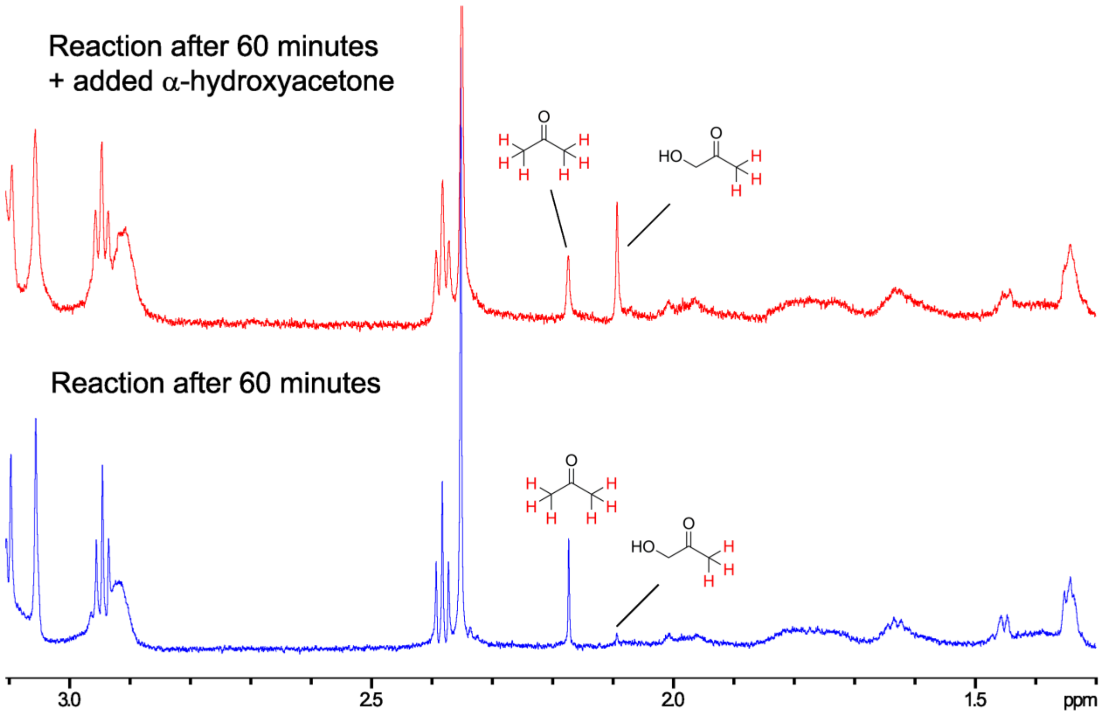

Figure S15 ^{1}H NMR spectrum of a sample containing the H3 Lysine-9(iPr) peptide and JMJD2E under standard conditions after 60 minutes at 25 °C and after addition of α-hydroxyacetone.

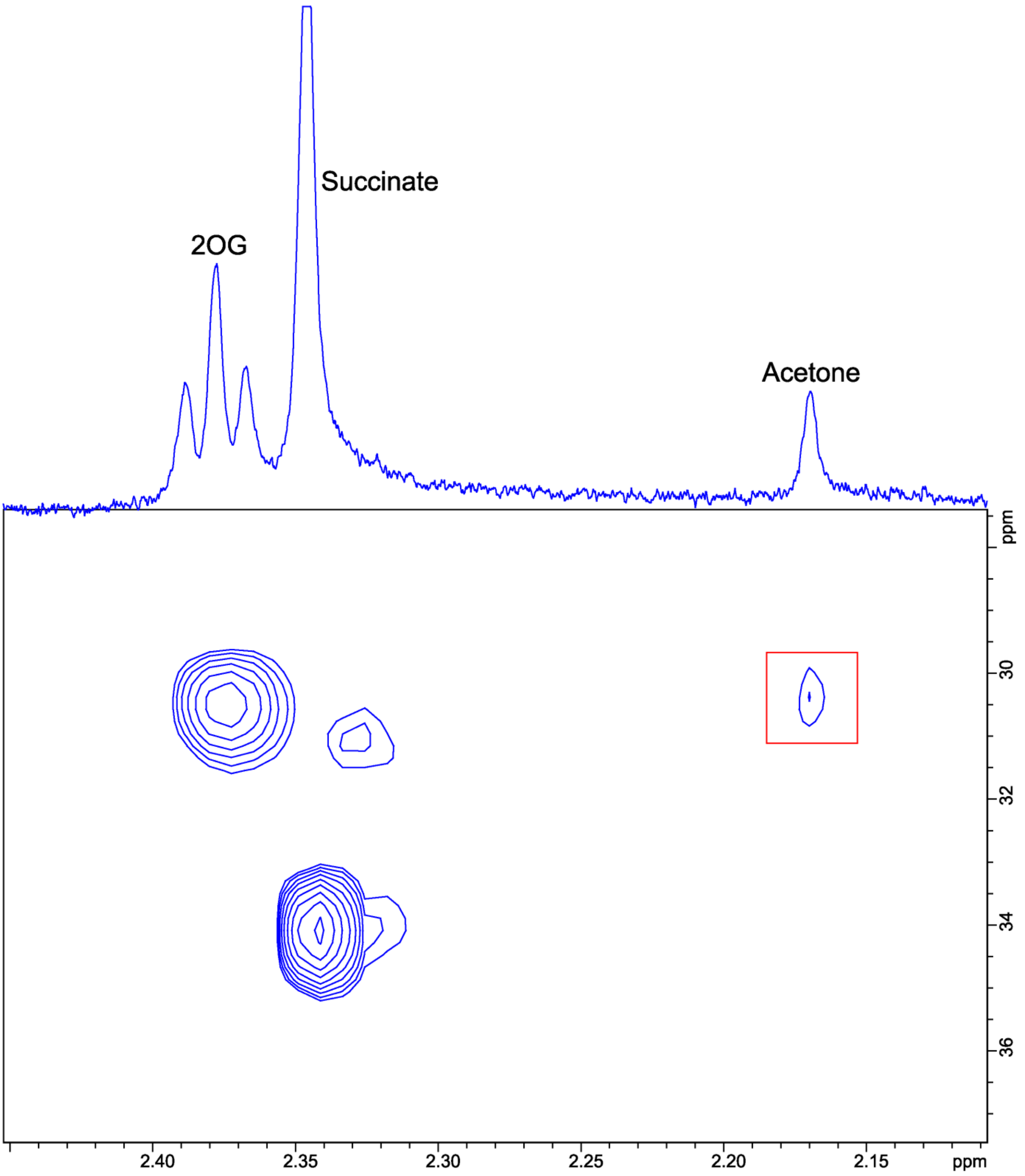

Figure S16 ^{1}H-^{13}C HSQC spectrum of of a sample containing the H3 Lysine-9(iPr) peptide and JMJD2E under standard conditions after 60 minutes at 25 °C. The ^{13}C chemical shift for acetone (at δ_C 30.4 ppm) is highlighted (red square).

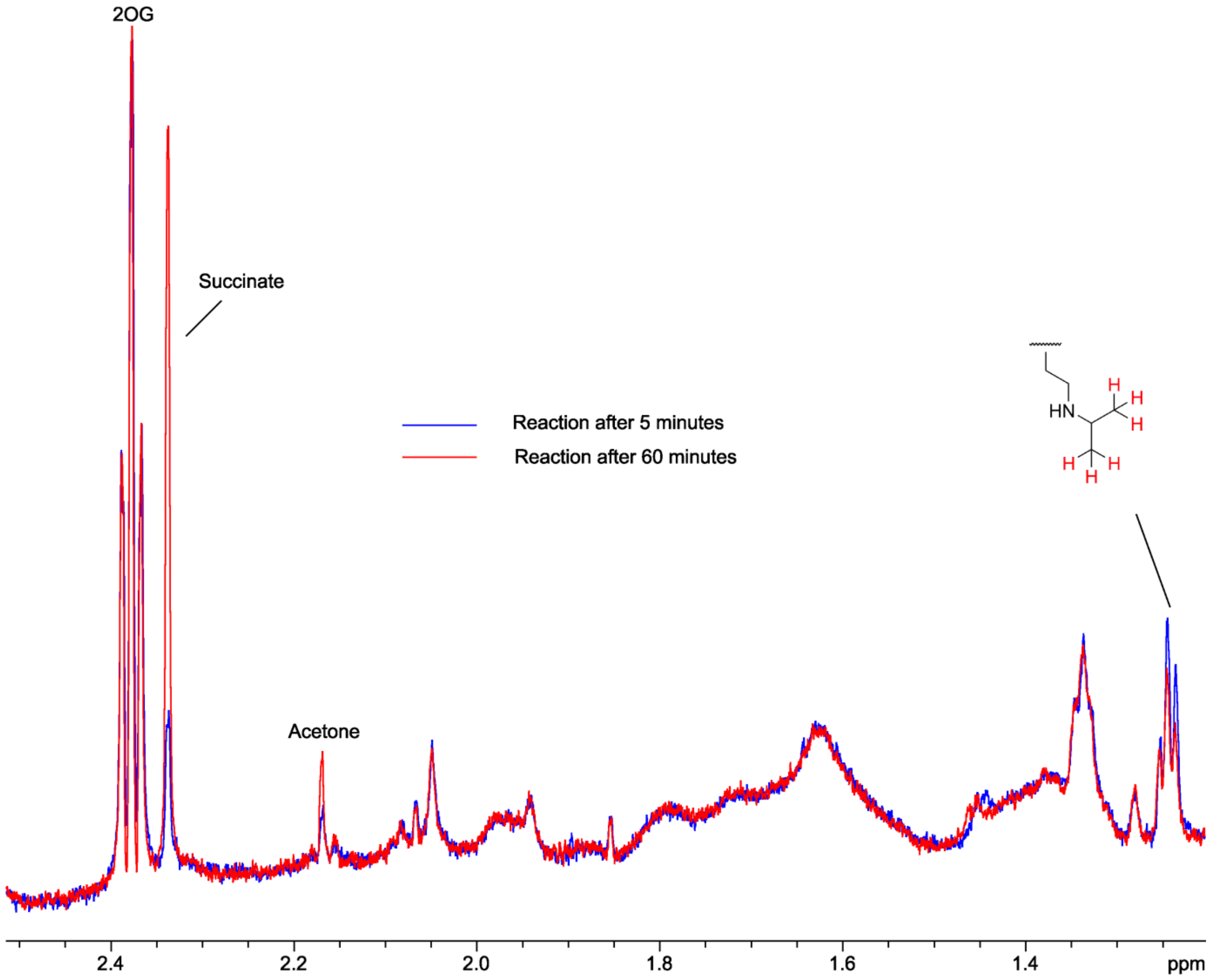

Figure S17 ^{1}H NMR spectra of a sample containing the H3 Lysine-9(iPr) peptide and PHF8 under standard conditions after 5 minutes (blue) and 60 minutes (red) at 25 °C. De-isopropylation is implied by the decrease in intensity of the doublet ^{1}H resonance at δ_H 1.26 ppm (indicating loss of the isopropyl group) and the increase in intensity of the singlet ^{1}H resonance at δ_H 2.17 ppm (indicating formation of acetone).

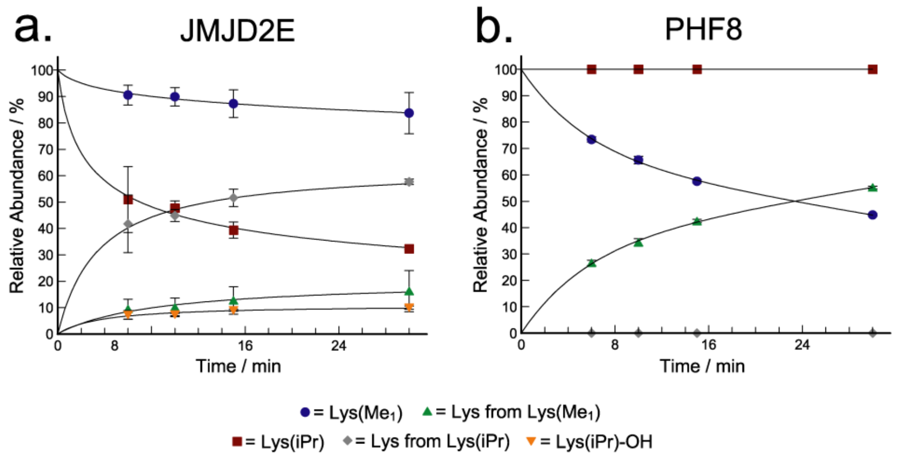

Figure S18 Competition experiments between the H3 Lysine-9(iPr) peptide and the H3 Lysine-9(Me_1) peptide with JMJD2E and PHF8. (a) Relative abundances of peptides over time in samples containing the H3 Lysine-9(iPr) peptide and the H3 Lysine-9(Me_1) peptide (sequence ART-Lys(Me_3)-QTAR-Lys(Me_1)-STGGK), in a 1:1 ratio, after incubation with JMJD2E at 25 °C. The Lys(iPr) peptide is de-alkylated more efficiently than the Lys(Me_1) peptide. b) Relative abundances of peptides over time in samples containing H3 Lysine-9(iPr) peptide and H3 Lysine-9(Me_1) peptide (sequence ART-Lys(Me_3)-QTAR-Lys(Me_1)-STGGK) in a 1:1 ratio, after incubation with PHF8 at 25 °C. Reaction of the Lys(iPr) peptide is strongly disfavoured (no de-isopropylation is observed) relative to the Lys(Me_1) peptide.

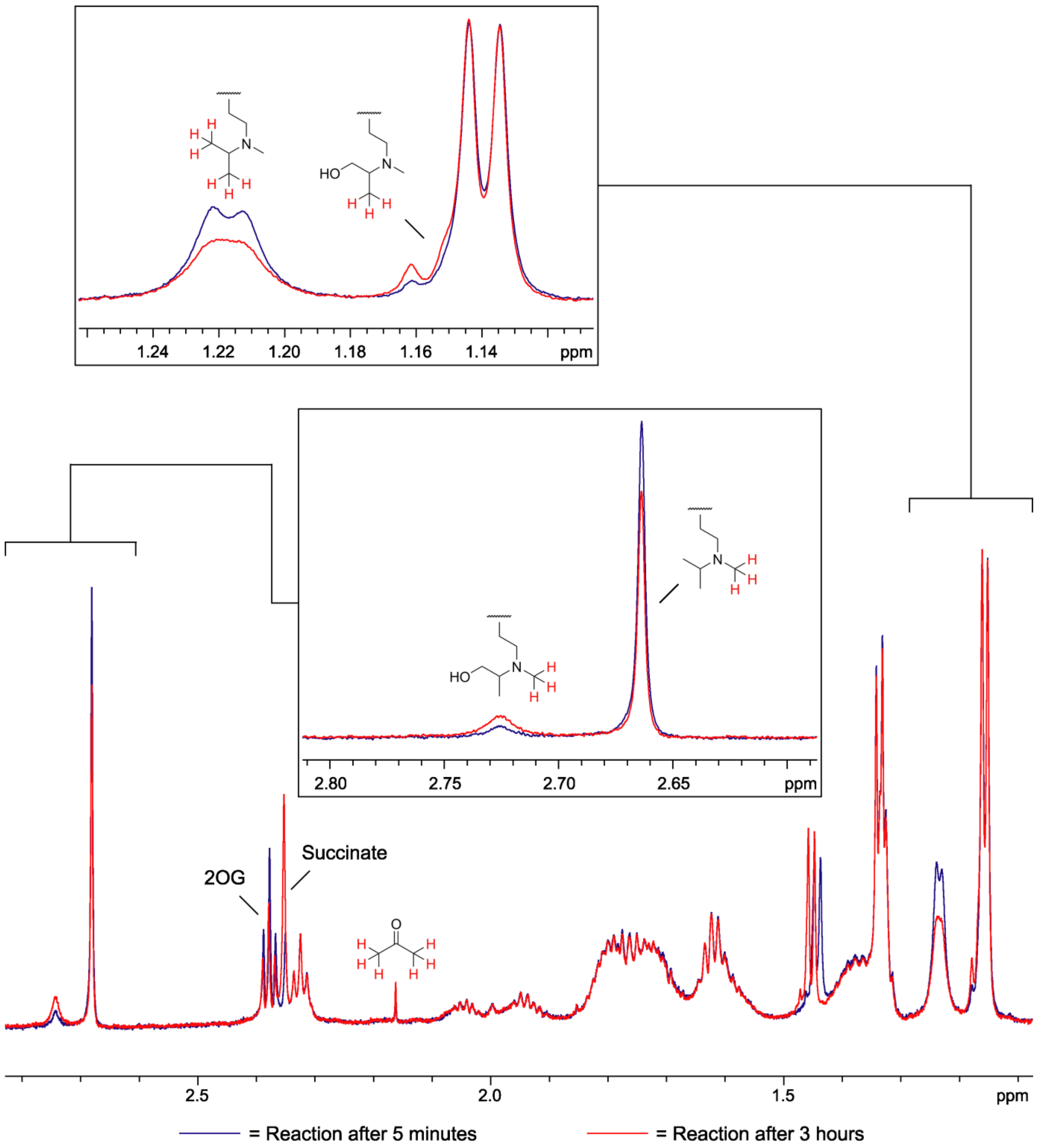

Figure S19 ^{1}H NMR spectra of a sample containing the H3 Lysine-9(Me/iPr) peptide (sequence ART-Lys(Me3)-QTAR-Lys(Me/iPr)-STGGKA) and JMJD2E under standard conditions after 5 minutes (blue) and 3 hours (red) at 25 °C. The resonance at δH 1.23 ppm decreases in intensity over the reaction period, indicating modification of the isopropyl group. A new resonance appears at δ_H 1.16 ppm, which was assigned to the hydroxylated product. Also, the resonance assigned to the substrate methyl protons (at δ_H 2.66 ppm) decreases in intensity and a resonance at δ_H 2.72 ppm increases in intensity. Formation of acetone (at δ_H 2.17 ppm) is observed.

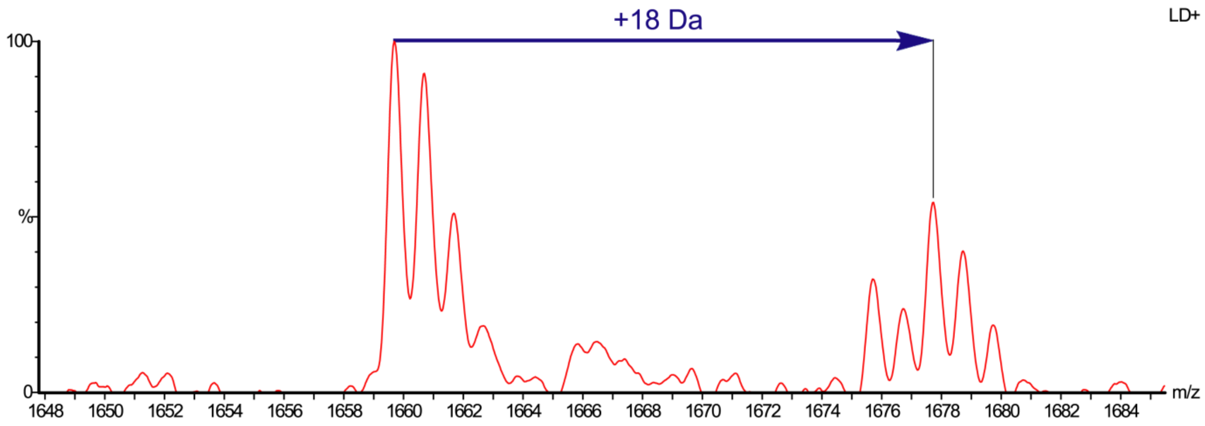

Figure S20 MALDI-TOF MS spectrum of a sample containing H3 Lysine-9(Me/iPr) peptide and JMJD2E under an $^{18}O_2$ atmosphere (after 3 hours at 25 °C). The presence of a +18 Da mass shift indicates incorporation of ^{18}O into the peptide. The presence of a +16 Da mass shift (suggesting some $^{16}O_2$ is present in the reaction mixture) may be due to incomplete degassing of the enzyme buffer.

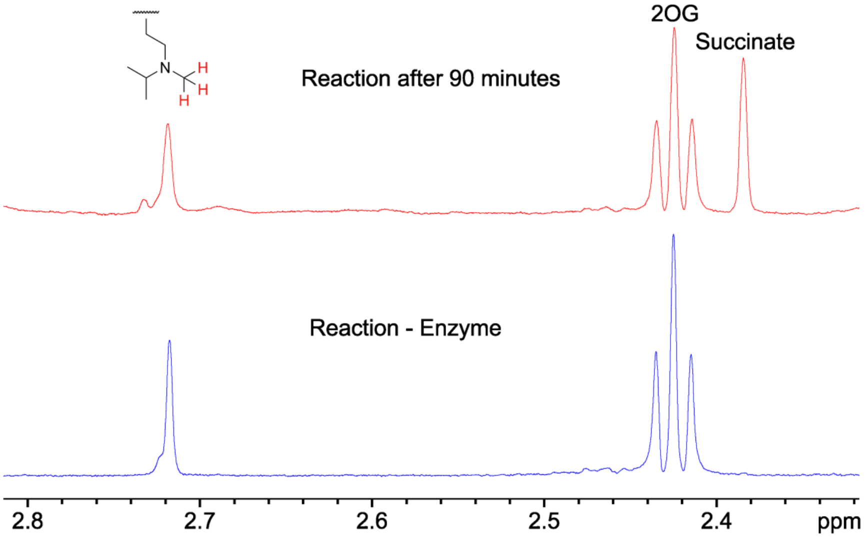

Figure S21 ^{1}H NMR spectrum of a sample containing H3 Lysin-36(Me/iPr) peptide (sequence PATGGV-Lys(Me/iPr)-KPHRY) and FBXL11 under standard conditions after 90 minutes at 25 °C. A spectrum after an identical incubation but without enzyme is shown below. Reduction of the singlet resonance at δ_H 2.72 ppm, reveals demethylation.

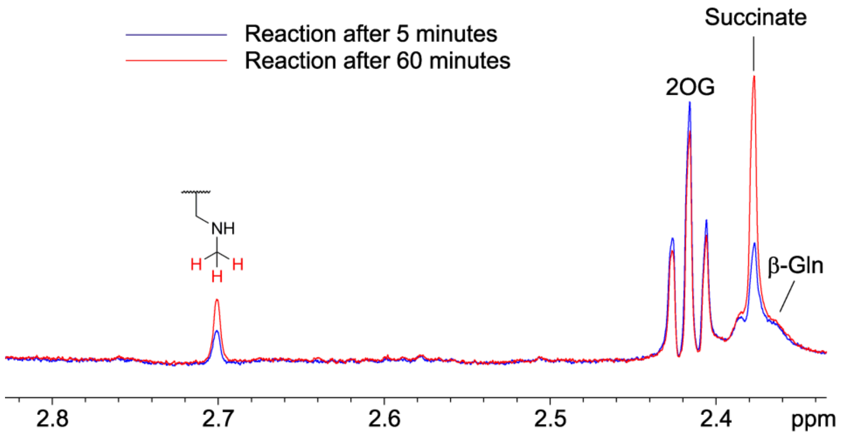

Figure S22 ^{1}H NMR spectrum of a sample containing H3 Ornithine-9(Me_2) peptide (sequence ART-Lys(Me_3)-QTAR-Orn(Me_2)-STGGKA) and PHF8 under standard conditions after 5 minutes (blue) and 60 minutes (red) at 25 °C. An increase in the intensity of the resonance at δ_H 2.70 ppm (assigned to the monomethylornithine methyl protons) is observed.

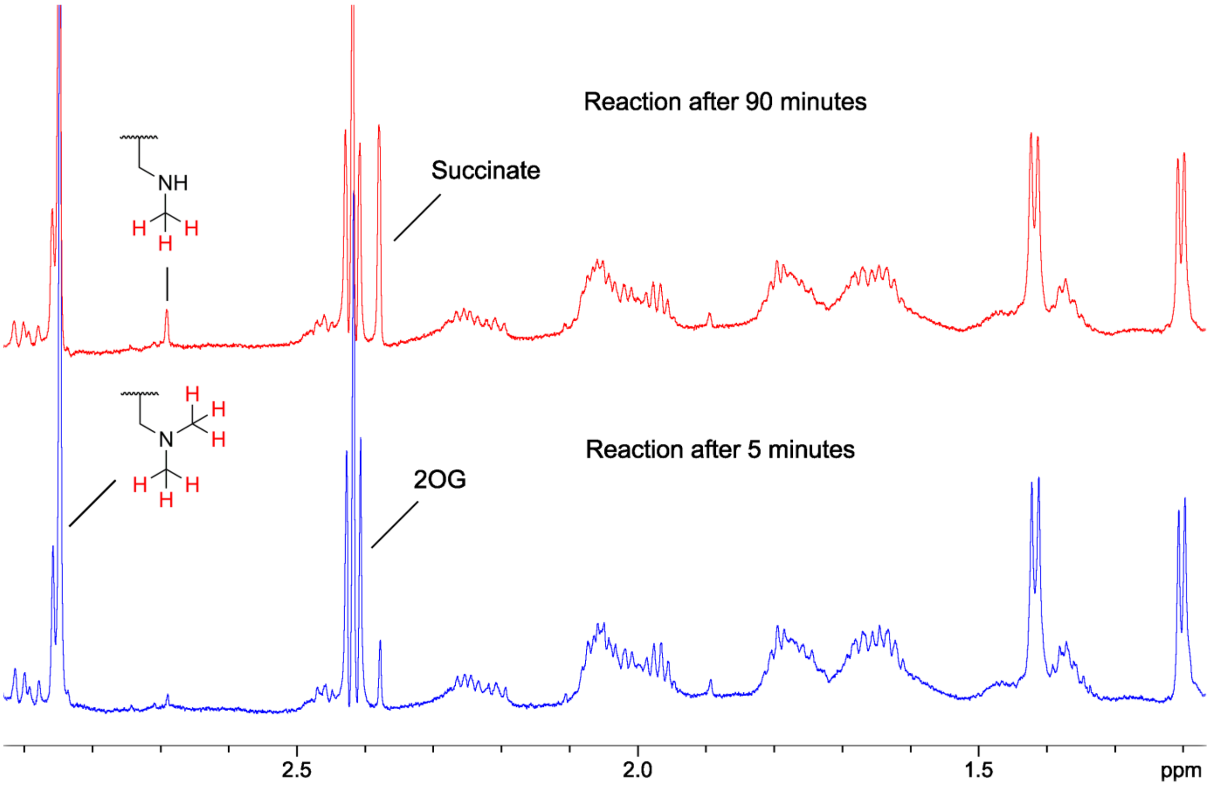

Figure S23 ^{1}H NMR spectrum of a sample containing H3 Ornithine-36(Me_2) peptide (sequence PATGGV-Orn(Me_2)-KPHRY) and FBXL11 under standard conditions after 5 minutes (blue) and 90 minutes (red) at 25 °C. An increase in intensity of the resonance at δ_H 2.68 ppm (assigned to the monomethylornithine protons) is observed.

Table S1 Relative reaction efficiencies of the lysine analogues with JMJD2E, PHF8 and FBXL11, as determined from the time course experiments. All peptides are weaker substrates than the corresponding Lys(Me_2) peptides (confirmed using pairwise competition experiments).

Demethylase	Lysine analogue
JMJD2E	Lys(Me/Et) > Lys(iPr) > Lys(Me/iPr)
PHF8	Lys(Me/Et) > Lys(iPr) > Orn(Me_2)
FBXL11	Lys(Me/Et) > Lys(Me/iPr) / Orn(Me_2)

Table S2 Kinetic parameters for reactions of lysine analogue peptides with JMJD2E.

H3 Peptide	K_M (μM)	V_{max} (μM s^{-1})	k_{cat} (s^{-1})
ARTK(Me_3)QTARK(Me_2)STGGKA	29.6±15.1	0.049±0.006	0.097±0.011
ARTK(Me_3)QTARK(Me/Et)STGGKA	42.7±13.8	0.052±0.005	0.104±0.009
ARTK(Me_3)QTARK(iPr)STGGKA	284.2±91.3	0.152±0.025	0.303±0.051
ARTK(Me_3)QTARK(Me/iPr)STGGKA	164.5±30.3	0.031±0.003	0.063±0.006

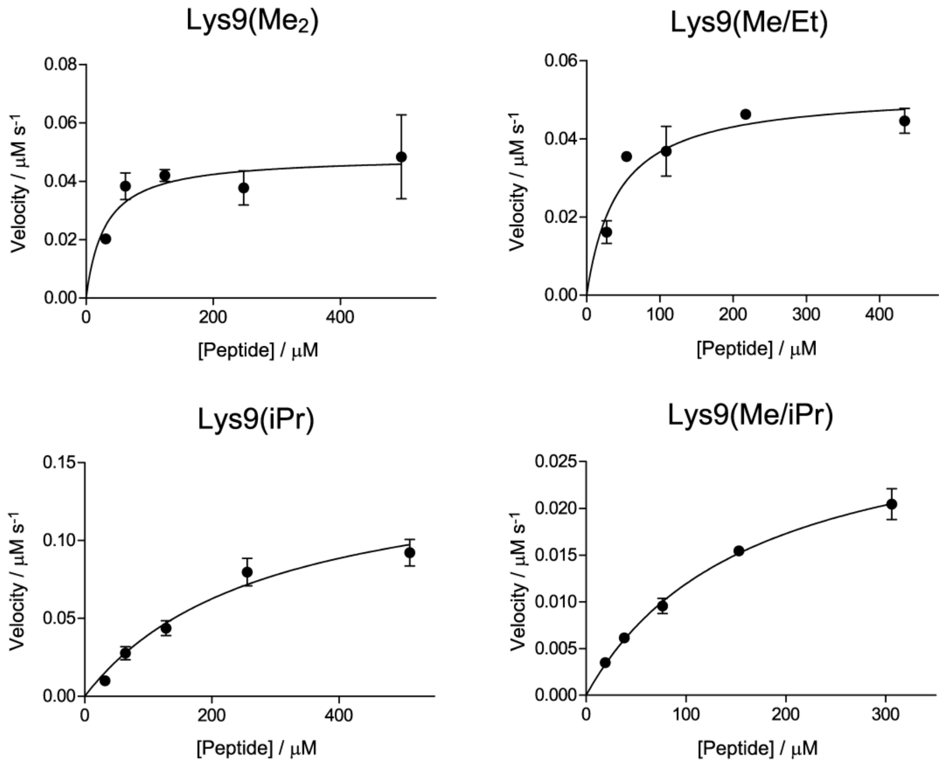

Figure S24 Michaelis-Menten curves for reactions of lysine analogue peptides with JMJD2E. Errors are given as standard errors of the mean.

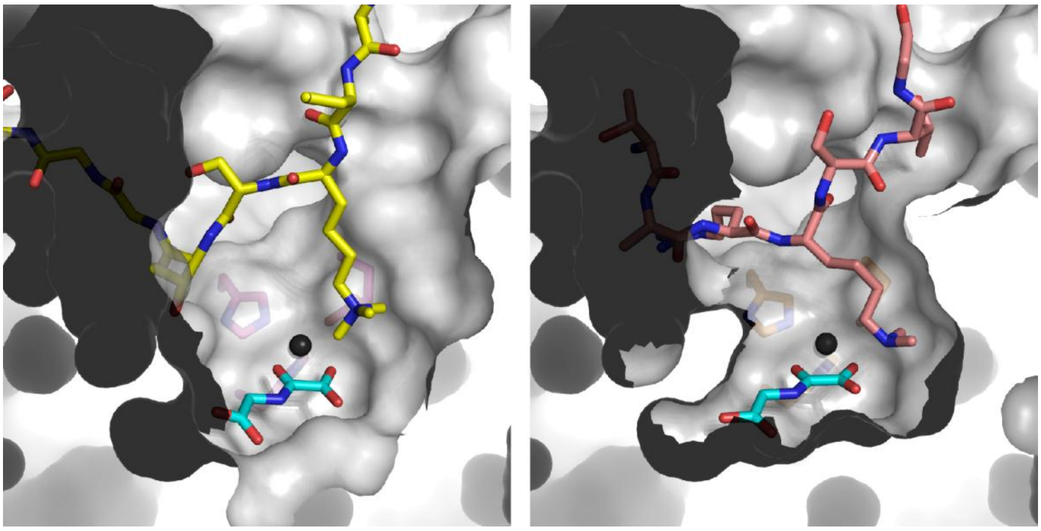

Figure S25 Views from two X-ray crystal structures of H3 lysine-9(Me_3) peptide (yellow) and H3 lysine-9(Me_2) peptide (pink) bound to JMJD2A (left, PDB ID: 2OQ6)[2] and PHF8 (right, PDB ID: 3KV4)[3] respectively. The methylated lysine penetrates deeper into the PHF8 active site than in JMJD2A. The catalytic iron is shown in black. The 2OG analogue *N*-oxalylglycine is shown in blue.

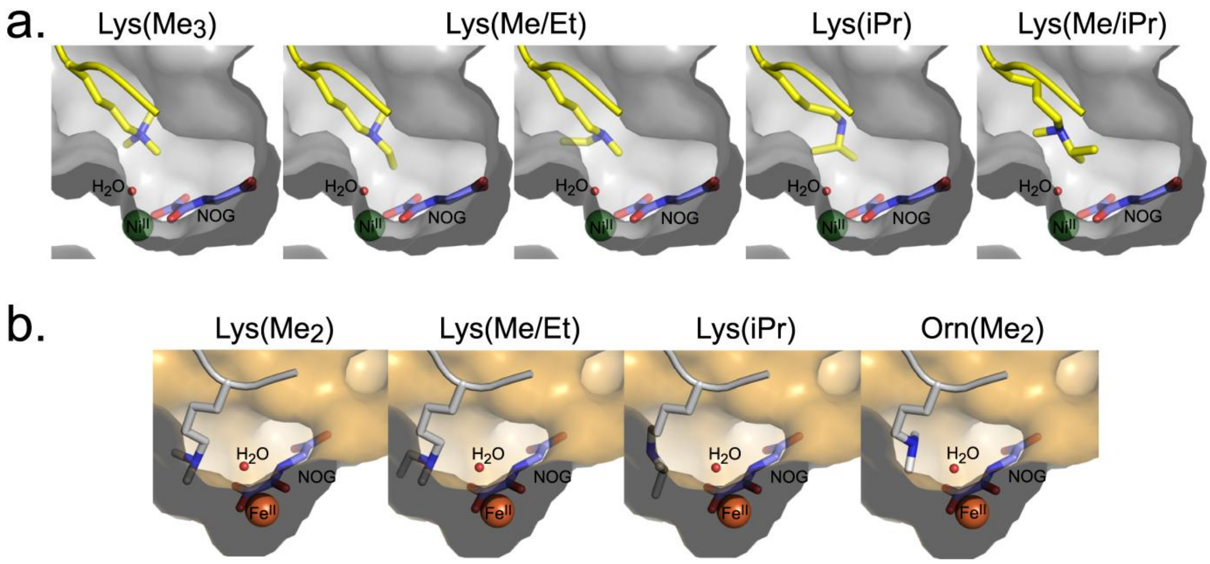

Figure S26 Manual docking of lysine analogues into demethylase active sites revals potential for accommodating alternative substrates. (a) Docking of Lys(Me/Et) (two conformations), Lys(iPr) and Lys(Me/iPr) residues into the active site of JMJD2A (a close structural homologue of JMJD2E, PDB ID: 2OQ6).[2] Lys(Me/Et) is both demethylated and de-ethylated by JMJD2E, implying the methyl and ethyl groups are positioned close to the catalytic iron. Note the Lys(iPr) residue, which is de-alkylated, can adopt a catalytically productive binding mode for this reaction. However, the Lys(Me/iPr) residue, which undergoes hydroxylation (with very little de-alkylation) is less likely to adopt a productive mode for de-alkylation due to steric constraints. (b) Docking of Lys(Me/Et), Lys(iPr) and Orn(Me$_2$) residues into the active site of PHF8 (PDB ID: 3KV4).[3] Lys(Me/Et) is not efficiently de-ethylated by PHF8, suggesting the ethyl group can only be positioned away from the catalytic iron. The Orn(Me$_2$) residues appear able to bind sufficiently close to the iron to enable hydroxylation of the methyl group. NOG corresponds to the 2OG analogue *N*-oxalylglycine. Note: the precise docking modes of the analogues should be regarded as speculative.

References

[1] R. J. Hopkinson, R. B. Hamed, N. R. Rose, T. D. W. Claridge, C. J. Schofield, *ChemBioChem*, **2010**, *11*, 506-510.

[2] S. S. Ng, K. L. Kavanagh, M. A. McDonough, D. Butler, E. S. Pilka, B. M. Lienard, J. E. Bray, P. Savitsky, C. Gileadi, F. Von Delft, N. R. Rose, J. Offer, J. C. Scheinost, T. Borowski, M. Sundstrom, C. J. Schofield, U. Oppermann, *Nature* **2007**, *448*, 811-816.

[3] J. R. Horton, A. K. Upadhyay, H. H. Qi, X. Zhang, Y. Shi, X. Cheng, *Nat. Struct. Mol. Biol.* **2010**, *17*, 38-43.
